# Supplementary material for: Visualization of associative exploration of temporal concepts via frequent patterns
Source: Patterns (N Y). 2025 Jun 11;6(8):101292. doi: 10.1016/j.patter.2025.101292 (PMC12365513; doi:10.1016/j.patter.2025.101292)
Supplement: Document S1. Figures S1–S3, Data S1, and Notes S1–S3 [file mmc1.pdf]

Patterns, Volume *6*

Supplemental information

Visualization of associative exploration of temporal concepts via frequent patterns

Tali Malenboim, Nir Grinberg, and Robert Moskovitch

Figure S1. Temporal Abstraction

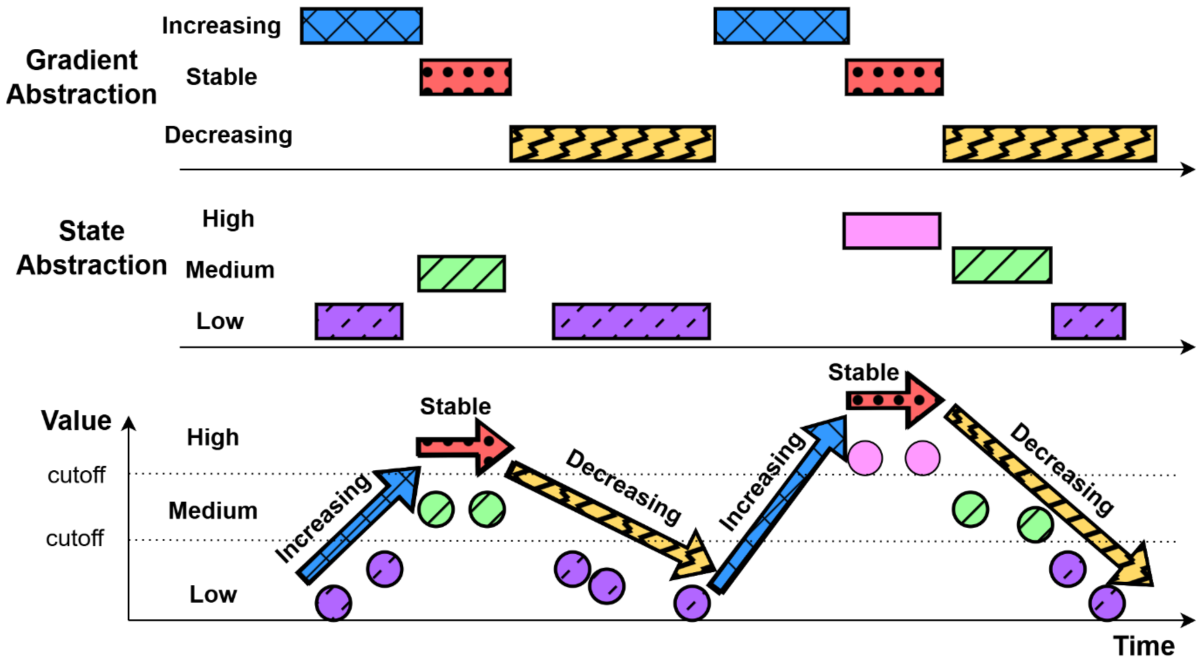

Raw time point values (at the bottom) are abstracted into symbolic time intervals series using State Abstraction (at the middle) with three state symbols (Low, Medium, High) and using Gradient Abstraction (at the top) with three gradient symbols (Increasing, Stable, Decreasing). The symbolic time intervals in the state abstraction chart correspond to the colored time point values (according to their value) in the bottom chart. The colored symbolic time intervals in the gradient abstraction chart correspond to the arrows that represent the gradients at the bottom chart.

Supplemental Note S1.

The process of transforming raw heterogeneous temporal data, such as time series, into symbolic time point series is called temporal abstraction (TA). Temporal abstraction encompasses various types, including state abstraction, gradient abstraction, SAX (Symbolic Aggregate approXimation), and more.

State Abstraction categorizes every time point value from the raw data to a state according to predefined cutoffs (such as low, medium or high glucose levels in blood tests), and later adjacent states having the same value are being concatenated into a symbolic time interval. Gradient Abstraction calculates the gradient of k recent time point values and concatenates the adjacent time points into a single STI which is labeled with the gradient (decreasing, stable or increasing).

For example, figure S1 demonstrates the process of two different TA methods on the same dataset. The chart at the bottom presents a univariate time series. The middle chart presents the state abstraction, applied on the dataset.

As can be seen, the first two time point values are categorized into the low state (since they are below the first cutoff), which is colored in purple. Since both values are in low state, they are concatenated into a symbolic time interval, which appears first in the (intermediate) state abstraction chart. Thus, the STIs in the intermediate chart are the state abstraction of the raw time point series at the bottom.

In the same manner, the top chart presents the result of applying the gradient abstraction of time point values at the bottom chart. The first blue arrow in the bottom chart describes the gradient of the first time point values, which is increasing, followed by a stable gradient colored in red. Looking at the top chart there are the corresponding STIs, having the first colored with blue for the increasing arrow, followed by the stable STI colored red, and so on.

Figure S2. TIRPs metrics example among three transactions

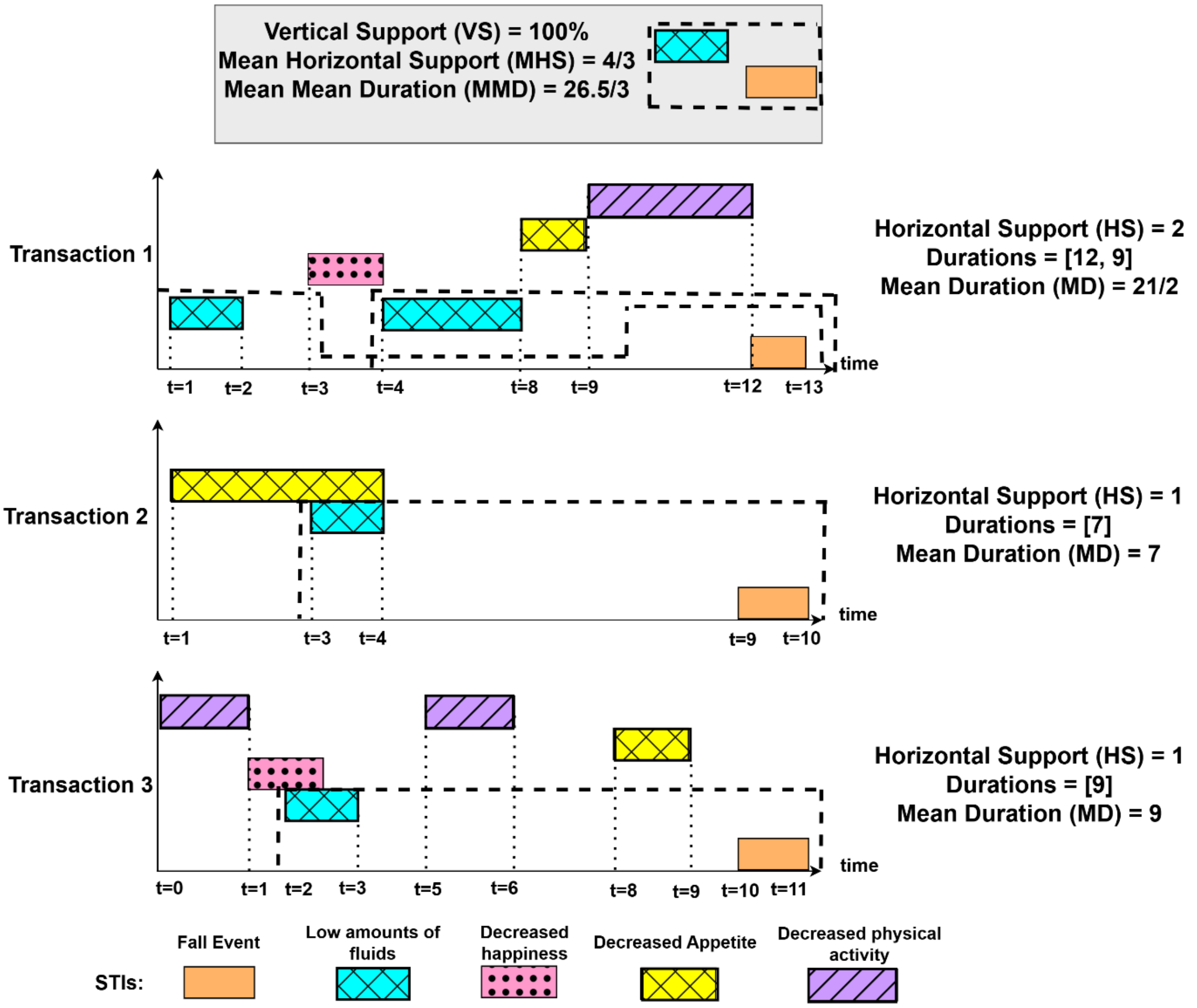

The explored TIRP is <'Low amounts of fluids, before a Fall Event'>, as illustrated in the gray rectangle in the top of the figure. The text on the right of every timeline, presents the TIRP's metrics in every supporting transaction. The overall TIRP's metrics are presented in the gray rectangle at the top.

Supplemental Note S2.

The output of a TIRPs mining algorithm is a set of all discovered frequent TIRPs.

TIRPs are commonly characterized by a number of metrics, that were explained briefly in the background section. Figure S2 shows an example of TIRP <'Low amounts of fluids, before a Fall Event'> and its metrics.

Every timeline represents the occurrences (instances) of the TIRP among its supporting transactions (transactions which the TIRP was discovered in). In addition to the TIRP's STIs, there are more STIs in the transactions, that are not part of this specific TIRP (such as 'Decreased Appetite'). The text on the right of every transaction's graph, presents the metrics of the TIRP in this specific transaction. For example, transaction 1, has two instances of <'Low amounts of fluids, before a Fall Event'> in the data, thus the Horizontal Support (HS) of this TIRP in transaction 1 is two. In contrast, the HS of this TIRP in transaction 2 and transaction 3 is one, only one single instance of this TIRP in these transactions. Thus, the Mean Horizontal Support (MHS) of this TIRP is the average of all the HS among the supporting transactions, which is 4/3.

The Vertical Support (VS) of this TIRP is 100%, because it appears in all the transactions in the dataset (given only these three transactions).

The Duration of a TIRP is set according to a specific instance of this TIRP in a specific transaction. For example, transaction 1 has two instances of TIRP <'Low amounts of fluids, before a Fall Event'> in the data, thus there are two durations in this transaction, which are 12 and 9. The first one starts in t=1 and ends with t=13, and the second starts in t=4 and ends with t=13. the Mean Duration (MD) in this transaction is the average of the TIRP's instances durations, which is 10.5. The Mean Mean Duration (MMD) is calculated as the average of all MDs of the TIRP, among all transactions, which is 26.5/3 in this example.

Figure S3, related to figure 7. Percentage of participants to answer exploration questions (y-axis) in a specific time range (x-axis).

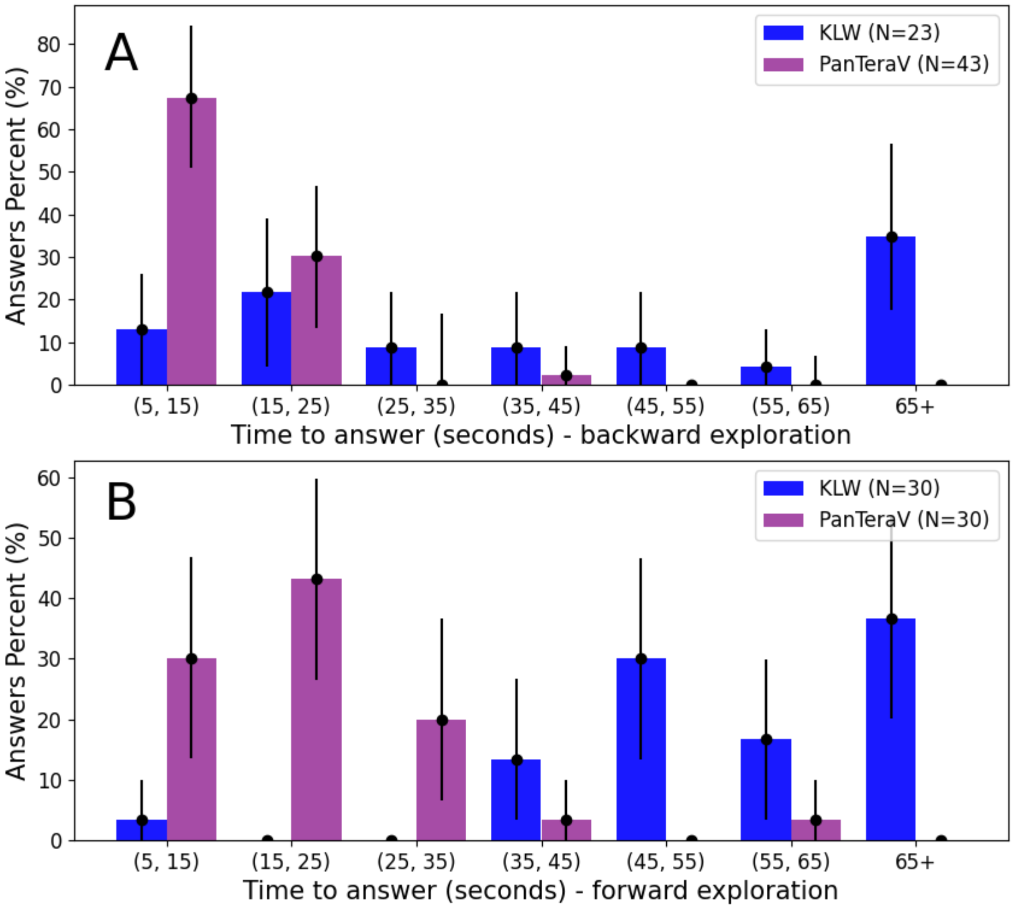

Panel A shows the results for backwards exploration questions, panel B for forward questions.

Supplemental Note S3.

In addition to figure 7, we show the results without cumulative calculation over time.

Figure S3 shows the percentage of correct answers versus the time it took participants to get them, both for backward (panel A) and forward (panel B) exploration.

As was mentioned in the results section, the number of answers reported is the number of participants that completed the tasks, after more than 5 seconds.

In about 35% of cases, participants who diligently attempted to answer the questions using the KLW interface,spent over 65 seconds on the task and often the full 90 seconds.

Yet, none of them were able to obtain the correct answer using the KLW interface.

In contrast, participants using the PanTeraV interface answered all of the questions correctly, and as shown in the figure, most of them took 5-15 seconds to get to the answer (67%) or between 15 to 25 seconds (30%).

Panel B of figure S3 shows the time it took participants to complete forward exploration tasks.

One can see that the vast majority of participants (a total of 95%) needed more than 35 seconds to complete the task using the KLW interface.

Using the same set of forward exploration questions, Panel B shows that most participants (~ 93%) using the PanTeraV interface took less than 35 seconds to complete the task.

In terms of accuracy, participants answered correctly at a high rate using both interfaces (98% for PanTeraV and 91% KLW interfaces) and those rates were not statistically different (P=0.17).

In summary, the results show that PanTeraV enables participants to tackle the same set of exploration questions significantly faster than using the KLW interface.

Data S1: user study questions divided to basic/exploration questions, per dataset and interface

Basic Questions - Diabetes Dataset

KLW Tabular baseline

What type of event reoccurs the most in the dataset (mean horizontal support)?

KLW Search baseline

Which age group is more likely to experience a period of an increase in HBA1C?

PanTeraV Tabular

What are the types of events that occur the most ACROSS patients (vertical support of at least 99)?

PanTeraV Graphical}

Which age group is more likely to experience a period of an increase in LDL?

Basic Questions - Falls Dataset

KLW Tabular baseline

What are the two types of events that reoccur the most in the dataset (mean horizontal support)?

KLW Search baseline}

Who is more likely to have an increased appetite, men or women?

PanTeraV Tabular

What are the types of events that occur the most ACROSS patients (vertical support of 100)?

PanTeraV Graphical

Who is more likely to be happier, men or women?

Backward Exploration Questions - Diabetes Dataset

KLW Tabular baseline

Researchers are looking for events that precede an increase in glucose level. They've identified that albumin often increases before glucose increases. Is there another type of event that precedes glucose increase, which is more frequent than the increase in albumin?

Researchers are looking for events that precede an increase in HBA1C levels. They've identified that cholesterol often increases before HBA1C increases. Is there another event type that precedes this sequence of increases (increase in cholesterol, before an increase in HBA1C)?

PanTeraV Tabular

Researchers are interested in the event types that precede an increase in Creatinine levels. They've identified that LDL often increases before Creatinine increases. Is there another event that precedes an increase in Creatinine, which is more frequent that LDL increase?

Researchers are looking for events that precede a period of increase in Cholesterol levels. They've identified that LDL often increases before Cholesterol increases. Is there another event type that precedes this sequence of increases (increase in LDL, before an increase in Cholesterol)?

Backward Exploration Questions - Falls Dataset

KLW Tabular baseline

Researchers are looking for events that precede decreased appetite. They've identified that falling often appears before decreased appetite. Is there another event type that precedes this sequence of events (falling, before decreased appetite)?

PanTeraV Tabular

Researchers are looking for events that precede decreased exercising. They've identified that falling often appears before the decrease in exercising. Is there another event type that precedes this sequence of events (falling, before less exercising)?

Forward Exploration Questions - Diabetes Dataset

KLW Search baseline

Is it more common that

a patient has an increase in HBA1C before an increase in Cholesterol?

a period of increase in HBA1C after a period of increase in Cholesterol?

PanTeraV Graphical

Is it more common that

a patient has a period of decrease in LDL before a period of decrease in glucose?

a period of decrease in LDL after a period of decrease in glucose?

Forward Exploration Questions - Falls Dataset

KLW Tabular baseline

Physicians want to examine the relationship between falling and attending fewer social events (recorded as 'Entertainment.Low'). In which of the following two patterns, the period of attending fewer social events is longer?

'low entertainment' then falling

falling then 'low entertainment'

KLW Search baseline

Is it more common that

a person falls and then engage less in entertainment

lose interest in entertainment and then fall

PanTeraV Tabular

Physicians want to examine the relationship between falling and decreased appetite (recorded as 'appetite.medium'). In which of the following two patterns, the period of decreased appetite is longer?

Decreased appetite then falling

Falling then decreased appetite

PanTeraV Graphical

Is it more common that

a person has an increased appetite before falling

after falling having an increased appetite
