# Supplementary material for: Visualization of associative exploration of temporal concepts via frequent patterns
Source: Patterns (N Y). 2025 Jun 11;6(8):101292. doi: 10.1016/j.patter.2025.101292 (PMC12365513; doi:10.1016/j.patter.2025.101292)
Supplement: Document S2. Article plus supplemental information [file mmc2.pdf]

Article

Patterns

Visualization of associative exploration of temporal concepts via frequent patterns

Highlights

- PanTeraV enables associative exploration of temporal patterns
- PanTeraV does not require prior knowledge in temporal data analysis
- PanTeraV supports diverse time-series types—continuous, discrete, and cross-domain

Authors

Tali Malenboim, Nir Grinberg, Robert Moskovitch

Correspondence

talimal@post.bgu.ac.il

In brief

This paper introduces PanTeraV, a visual interface for exploring temporal patterns, both continuous and discrete, independent of the mining algorithm. By enabling bidirectional exploration of patterns with an efficient indexing approach, PanTeraV significantly advances the discovery of complex temporal-interval-related patterns (TIRPs). A usability study highlights that PanTeraV allows faster, more accurate pattern discovery compared to existing tools, offering a powerful solution that does not require prior data mining expertise, broadening accessibility for diverse users.

Malenboim et al., 2025, Patterns 6, 101292
August 8, 2025 © 2025 The Author(s). Published by Elsevier Inc.
https://doi.org/10.1016/j.patter.2025.101292

CellPress

Patterns

CellPress
OPEN ACCESS

Article

Visualization of associative exploration of temporal concepts via frequent patterns

Tali Malenboim,[1,2,*] Nir Grinberg,[1] and Robert Moskovitch[1]
[1]Software and Information Systems Engineering, Ben-Gurion University of the Negev, Beer Sheva 8410501, Israel
[2]Lead contact
*Correspondence: talimal@post.bgu.ac.il
https://doi.org/10.1016/j.patter.2025.101292

THE BIGGER PICTURE Understanding patterns in data that unfold over time can reveal valuable insights about cause and effect, trends, and hidden relationships. However, analyzing these patterns is often complex and overwhelming because there are countless ways events can be connected across time. Traditional tools usually focus on examining one pattern at a time, making it hard for analysts to see the bigger picture or discover new connections. PanTeraV is a user-friendly visual tool designed to help researchers and data scientists explore large collections of time-based patterns more effectively. It allows users to look both forward and backward in time from a specific pattern, revealing what tends to happen before or after an event. This bidirectional approach helps uncover patterns that might not be obvious with conventional methods, such as early signs that lead to a particular outcome or behaviors that follow an initial event.

SUMMARY

Most studies on temporal pattern visualization have focused on a single pattern and its metrics and supporting instances. However, the output of a mining process is typically an enumeration tree of frequent temporal patterns. A key challenge is exploring these patterns to identify those of interest for an expert or data scientist. Recently, it was suggested that the enumeration tree be browsed from the root downward through extended patterns. We introduce PanTeraV, a visualization system for statistical and analytical exploration of a large enumeration tree of complex temporal patterns. Demonstrated with time-interval-related patterns (TIRPs), it enables bidirectional exploration based on user-selected symbolic time intervals. The system consists of two visualizations: tabular, for navigating symbolic time intervals, and graphical, which presents relevant patterns in a bubble chart encoding multiple metrics. A user study on two real-world datasets shows that PanTeraV enables faster exploration of temporal patterns and allows users to discover associations of symbolic time intervals that were previously inaccessible.

INTRODUCTION

Heterogeneous longitudinal data analysis is a meaningful challenge in various domains, such as healthcare, manufacturing, cyber security, and more. Temporal knowledge discovery has the ability to advance our understanding of the way events occur over time and evolve dynamically. Over the past decades meaningful advancements were made in the discovery of frequent temporal patterns.[1–5] In the process of temporal knowledge discovery, frequent temporal patterns are discovered,[6,7] which typically describe a common behavior of a subset of transactions (i.e., patients' data) of a mined population. This provides great opportunities to acquire meaningful, ideally actionable, temporal knowledge. However, one of the major challenges in the process of knowledge discovery is the often vast number of frequent temporal patterns discovered. The number of discovered patterns is not necessarily a direct result of the size of the dataset but rather depends on the parameters used in the pattern mining algorithm, primarily the minimal support (the minimal number of transactions for a pattern to be considered frequent), but there can be other parameters as well. Thus, even for relatively small datasets, the output can contain hundreds or even thousands of patterns, especially in low levels of minimal support, as we elaborate later. Being able to explore and get to the patterns of interest is a challenge that we focus on in this paper. For that purpose, visualization is essential and can be useful.

In the past decades, most related works focused on the visualization of longitudinal data for reasoning purposes[8–10] or visualization of sequential patterns (patterns that consist of events, or concepts, with no duration).[11–16] However, those studies focused on the visualization of the patterns and their properties, as well as their supporting instances (instances of discovered

This is an open access article under the CC BY license (http://creativecommons.org/licenses/by/4.0/).

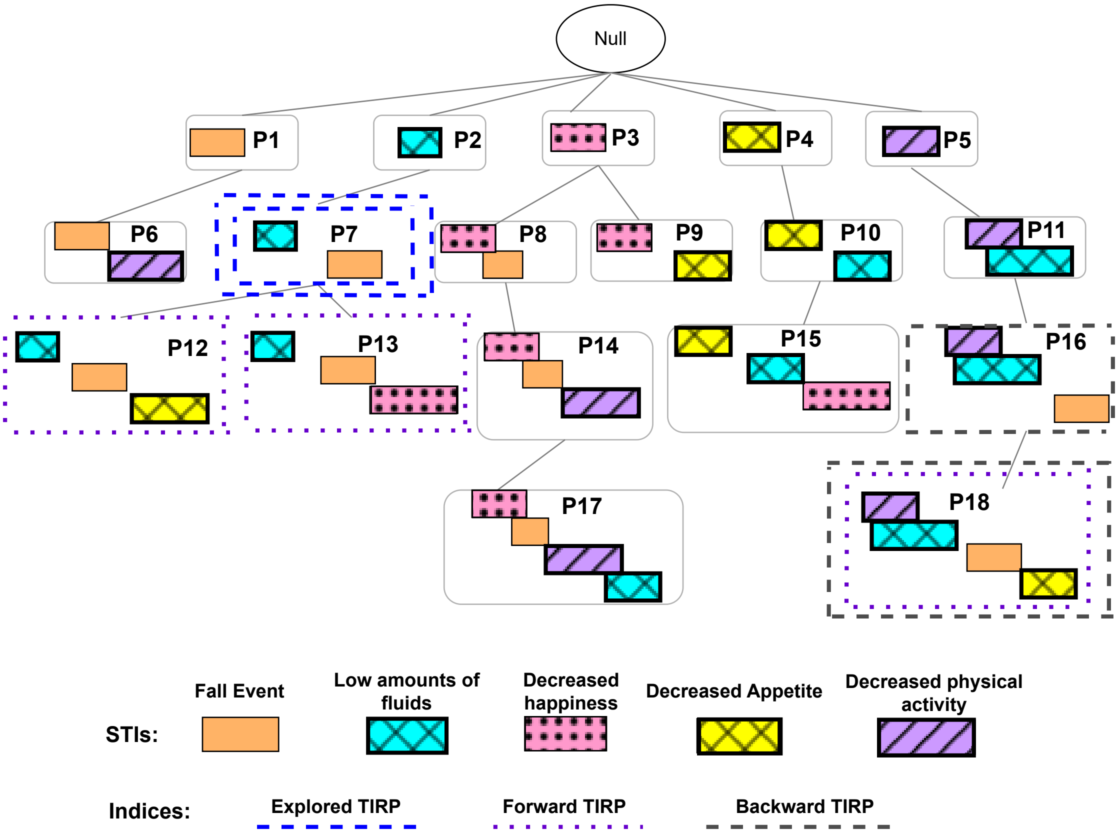

Figure 1. TIRP enumeration tree resulting typically from a mining process

The first level contains frequent TIRPs with a single STI (one-sized TIRP). Each of them is the root of a branch (or a tree) of all the TIRPs, starting with the symbol of the STI at the root. Further down the pathways are extended TIRPs, having an additional STI at each lower level extending the TIRP above (for example, P12 is an extension of P7). Patterns P12, P13, P15, P17, and P18 do not have extended TIRPs, since their extended TIRPs were not found to be frequent (above the minimal vertical support). TIRP P7 (double dashed in blue) represents an example of a TIRP that the user chose to explore. TIRPS P12, P13, and P18 (dotted in purple) are possible TIRPs to extend P7 because they consist of P7's STIs and additional STIs afterward. P16 and P18 (dashed in gray) are possible backward extensions of P7 because they consist of all P7's STIs and additional STIs beforehand. Further explanation regarding TIRP extensions (both forward and backward) will be discussed in the methods.

patterns within a transaction), rather than on exploring and acquiring the patterns of interest from a collection of discovered temporal patterns.

In this paper, while we introduce a visualization methodology for the exploration of generally temporal patterns, we demonstrate it on the use of time-interval-related patterns (TIRPs), since they can be discovered from heterogeneous multivariate temporal data and can be used in various types of temporal variables, whether sampled regularly or irregularly; event series; or events that may have varying durations.[3] Using temporal abstraction (TA), various forms of temporal variables can be transformed into a series of meaningful symbolic time intervals (STIs; i.e., periods of time in which the variable is in a specific state or is increasing/stable/decreasing, etc.). From such a database of transactions of STI series, TIRPs can be mined.[17] We further elaborate on the process of temporal abstraction and TIRP mining in the next section; however, typically, the output of a pattern discovery process is an enumeration tree of all the frequent patterns that were discovered. Such an enumeration tree, like the one illustrated in Figure 1, may include hundreds or even tens of thousands of patterns from which knowledge should be acquired. So far, only one interface has been introduced in the literature, KarmaLegoWeb (KLW), for this purpose, which allows exploration of TIRPs.[18] KLW was designed to enable an insightful exploration of the pattern tree. It enables browsing the pattern tree from the root to the leaves along a specific branch in the enumeration tree of patterns. Additionally, it enables one to retrieve patterns according to a query specifying the components of the pattern of interest, such as the first, intermediate, or last, as we describe in Figure 2.

Figure 2 demonstrates one of the exploration techniques in KLW, given the intention of looking for patterns that contain event <"Low amounts of fluids" before "Fall event"> and to explore which events appear beforehand (Figure 2A). Figure 2B shows how it is possible to query and, by that, filter the suitable patterns according to the first, intermediate, and last events in them. Thus, the only option to query, given the current need, is setting the last event to <"Fall event"> but, since it is not possible to query the event that appears immediately beforehand, setting the intermediate event (which can be

CellPress
OPEN ACCESS

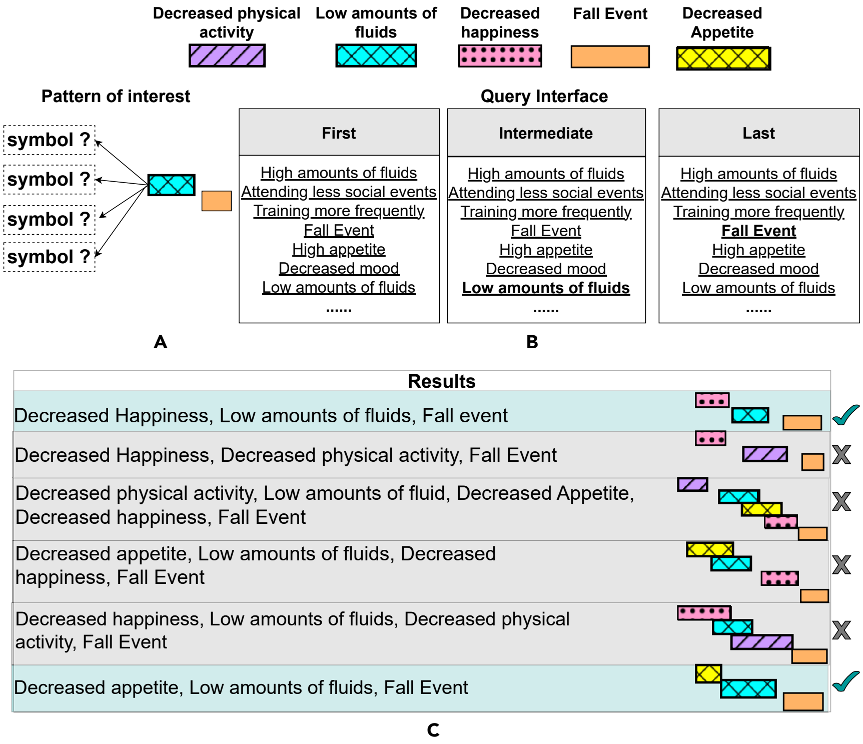

Figure 2. Limited exploration through querying of enumeration tree of frequent TIRPs in KLW

(A and B) A pattern of interest (A), <"Low amounts of fluids" before "Fall event">, that the user wants to further explore is presented. The user is curious of what precedes "Low amounts of fluids" (question marks). For that, (B) shows the query interface of KLW that offers to specify the first, intermediate, or last symbols of a pattern of interest. The user can specify <"Low amounts of fluids"> as the first STI and <"Fall event"> as the last STI (appear in bold).

(C) The results of the specified query. Every row is a pattern (visualized on the right) described in the text. Blue rows match the relevant patterns from (A), while gray rows are patterns that match the query in (B) but not what the user expected in (A). This figure and the next examples consist of events and patterns from the falls dataset, which we used in the user study.

also two events earlier) to "Low amounts of fluids." The first event can be left not set, as shown in the figure. The result of such query (Figure 2C) is a list of patterns that end with < "Fall event"> and in which the event <"Low amounts of fluids"> appears as well, not necessarily immediately beforehand. Thus, the long list shown partially in Figure 2C includes patterns of which only part is relevant. The blue rows correspond to patterns that match the need in Figure 2A, while the gray rows are patterns that match the query in Figure 2B but not the user's need (Figure 2A).

In order to enable associative exploration through discovered frequent patterns and, by that, provide the user an intuitive exploration process, we implement an innovative querying interface that addresses the user's needs and is also more efficient.

PanTeraV's innovation can be mainly described in the two following aspects. First, it introduces an efficient bidirectional indexing algorithm, which enables users to explore patterns, while providing immediate access to patterns that cannot be discovered using existing visualization tools (such as KLW). This introduces an advancing pattern exploration technique, which can benefit domain experts and reveal new unreachable patterns. Second, by offering an intuitive and user-friendly interface, PanTeraV intends to help data scientists, or domain experts, to derive conclusions faster and, more importantly, that are actionable as a result.

We demonstrate PanTeraV's usability on two real-world datasets and analyze the interface's performance in terms of the average time duration to complete a task and its accuracy.

The main contributions of this paper are as follows.

(1) It describes a visualization approach for associative exploration of temporal events through frequent patterns.
(2) It provides an efficient indexing technique that allows further exploration and investigation based on the enumeration tree of frequent temporal patterns.
(3) It presents a rigorous empirical evaluation of the interfaces using two real-life datasets and two different user groups.

RESULTS

Backward and forward exploration questions

Figure 3 shows the cumulative percentage of correct answers versus the time it took participants to get them, both for backward (solid line) and for forward (dashed line) exploration questions. Using KLW, in 22 backward questions, participants understood within 5 s that it would take them far more than the allotted time (90 s) to answer the question and decided to skip to the next question. To make a fair comparison with the PanTeraV interfaces, we excluded similarly "quick" answers from the PanTeraV interfaces ($N = 2$), although the results were not fundamentally different when including them. Thus, for the backward questions in KLW there are 23 answers and for PanTeraV's backward questions 43 answers.

Most of the participants (≈70%) took less than 15 s to answer the backward questions using PanTeraV interfaces, while 52% of participants took 45 s to realize they could not answer these questions using KLW. In about 35% of cases, participants spent over 65 s on the task and often the full 90 s. Yet, none of them were able to obtain the correct answer using the KLW interface. All participants managed to complete the backward questions using PanTeraV and none of them using KLW. The dashed lines show the time it took participants to complete forward exploration

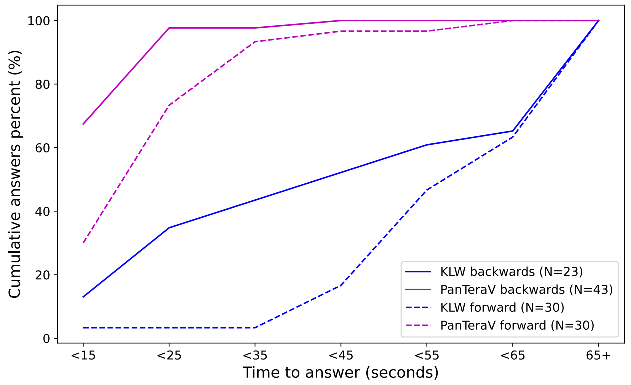

Figure 3. Cumulative percentage of participants' answers to complete the backward or forward exploration tasks (*y* axis) until a specific time range (*x* axis)

Pink lines describe the cumulative answers of participants using PanTeraV, while blue lines correspond to the KLW baseline. Dashed lines describe results for forward exploration questions, and solid lines describe results for backward exploration tasks.

tasks. One can see that the vast majority of participants (a total of 95%) needed more than 35 s to complete the task using the KLW interface. Using the same set of forward exploration questions, most participants (≈93%) using the PanTeraV interface took less than 35 s to complete the task. Specifically, most participants (73%) took less than 25 s to complete these tasks. In terms of accuracy, participants answered correctly at a high rate using both interfaces (98% for PanTeraV and 91% KLW interfaces), and those rates were not statistically different ($p = 0.17$). Note that supplemental results can be found in Figure S3.

In summary, the results show that PanTeraV enables participants to tackle the same set of exploration questions significantly faster than using the KLW interface ($p < 0.001$ for both forward and backward exploration). Regarding backward exploration questions, as was previously mentioned, KLW cannot be used to answer these questions in a reasonable time; hence, its accuracy was 0% in this type of question. Conversely, PanTeraV achieved a perfect score of 100% accuracy in backward exploration tasks. Therefore, we found significant gains in using PanTeraV and significantly larger gains when tackling backward exploration questions, which were hard, prone to error, and very time consuming using the KLW interface.

Basic usability questions

To make sure that the added capabilities of PanTeraV do not come at the expense of usability in simple tasks, we examined the response time and accuracy when performing basic usability tasks. For example, a basic task is to find the most frequent TIRP of size 1.

We found that participants answered correctly all eight basic tasks when using both the KLW and the PanTeraV interfaces. In terms of time duration, it took participants 11.18 s on average to answer these questions using the KLW interface and 11.66 s on average to answer them using PanTeraV. The difference was not statistically significant ($p = 0.28$). Therefore, we conclude that are no significant differences between the interfaces in performing basic tasks.

Dependence on prior knowledge in TIRPs

Here, we examine whether there are performance differences between participants depending on their prior knowledge of TIRP mining. To that end, we compared the time it took participants with prior knowledge of TIRP mining versus those without it. It is important to note that all participants had some background in data science, and therefore the comparison group is referred to as Data Science.

Figure 4 shows the mean time *difference* between the groups (*x* axis), where higher positive values represent longer completion times for the TIRP Mining knowledge group. The differences are shown for both the diabetes and the falls datasets, using 95% bootstrapped confidence intervals. Figure 4, left, corresponds to backward exploration questions, and the right corresponds to the forward exploration questions. The red horizontal line represents the zero line of no difference between groups. The results in Figure 4 show that there are no significant differences between the times it took each of the groups to complete the task, since the zero line is well contained within the confidence intervals for both datasets ($p > 0.05$). Furthermore, the confidence interval is smaller for the backward exploration questions compared to the forward exploration questions.

This suggests that PanTeraV enables participants to complete backward exploration tasks more consistently, irrespective of prior knowledge. We also note that the confidence intervals are smaller in the diabetes dataset compared to the falls dataset, which is consistent with the smaller number of STIs in the diabetes dataset. Thus, these results show that there are no significant differences in performance that depend on having prior knowledge of TIRP mining, neither in forward nor in backward exploration tasks.

DISCUSSION

This paper introduces PanTeraV, a visual interface designed to support the exploration of temporal concepts, which we defined more technically as STIs. The exploration of the associations of the various temporal concepts consists in an enumeration tree of frequent temporal patterns that were discovered by a mining process. Unlike previous studies that focused on the visualization of a specific temporal pattern and its properties, its supporting instances, or their appearance in the raw temporal data, PanTeraV focuses on the visualization of an enumeration tree of TIRPs, which is its input rather than the raw temporal data from which the patterns were mined. PanTeraV is demonstrated with TIRPs, consisting in Allen's seven temporal relations, while other versions of temporal relations can be used.[19,20] Moreover, the visualization methods of PanTeraV can potentially also work with other types of temporal patterns, such as sequential patterns, which we intend to do in the future. The main purpose of PanTeraV is to enable a user to explore the STIs and the patterns associatively (if the user is interested in knowing which STIs are earlier or later than the current STI in focus, she can easily see that according to the relevant patterns). The PanTeraV interface is composed of two types of visualizations, tabular and graphical, each with its own advantages. While the tabular view provides a more informative display, the graphical view shows a bigger view of the patterns and their distribution over various metrics. We presented a comprehensive usability study of

CellPress
OPEN ACCESS

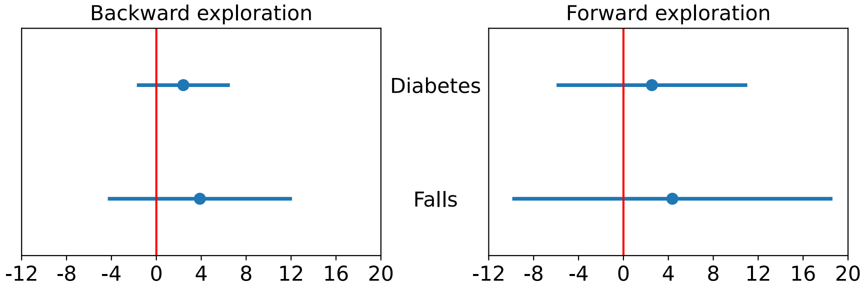

Figure 4. Time differences between students with prior knowledge of TIRP mining and students with basic understanding of data science

The *x* axis presents the mean time difference between the groups. The *y* axis presents the two datasets tested in the user study. The vertical red line is placed at zero, representing no difference in order to emphasize statistical significance. $p > 0.05$.

PanTeraV interfaces in comparison to a relevant baseline.[18] The user study showed that PanTeraV enables the exploration of complex TIRPs rather faster and more accurately than the baseline. Moreover, the interface does not require prior knowledge of the methodology and can be used after a short explanation of the methodology and interfaces.

In addition, although the interface's main concern is enabling the exploration of complex temporal patterns, PanTeraV can also be used to perform simple forward exploration tasks that do not involve backward exploration.

Limitations of the study

While PanTeraV demonstrates a unique approach in the visualization of associative exploration of STIs based on frequent temporal patterns, the limitations of the study should be discussed. First, the user study included 15 participants. While this is a common number of participants in such user studies, it may sound limited. However, note that there were a sufficient number of participants to get statistically significant results.

PanTeraV was evaluated on two datasets that are different in the heterogeneity of the temporal data. It could be better to have more datasets, although PanTeraV is generic and not domain dependent, since it is overwhelming for users to experiment with more than two datasets. However, while those two datasets come from different domains, one a clinical dataset of diabetic patients and another that describes the daily activities of residents of care homes (not clinical data), the main difference between these two datasets is the nature of the data. While the diabetes dataset includes regularly (monthly) sampled data, the falls dataset includes irregularly logged daily activities that may contain continuous or discrete measurements. While it would be good to demonstrate PanTeraV on other domains of data, it is important to highlight that PanTeraV was designed to enable exploration of TIRPs and STIs' associations through visualization, and it is not limited to a specific domain nor whether it is regularly or irregularly sampled due to the use of temporal abstraction.

Moreover, the user study did not intend to evaluate PanTeraV's usability in the context of a specific domain. While it is out of the scope and context of the purpose of PanTeraV, it seems desirable to further the use of PanTeraV to enable showing specific instances of TIRPs in the raw data,[21] which we intend to do in future work.

Background

Temporal knowledge acquisition from an enumeration tree of frequent temporal patterns through effective visualization is the focus of this paper. It is demonstrated with TIRPs, for which we provide a background here.

We start by introducing temporal abstraction and its use in overcoming challenges with heterogeneous temporal data. Afterward, we go over knowledge discovery algorithms for TIRPs, which are the focus of this paper, and finally we refer to relevant temporal data and temporal patterns' visualization interfaces in the literature.

Temporal abstraction

A major challenge in temporal data is the heterogeneity of the sampling forms and the representation of the data, which can be time-point series sampled regularly or not or events with or without duration. A common approach to address these issues is to use temporal abstraction, which transforms time-point series into meaningful STI series representations, hence enabling a uniform representation of the heterogeneous temporal variables. Thus, if the dataset consists of temporal variables with different time granularity, the process of temporal abstraction handles these differences and converts the dataset into a united representation of STIs. Several common temporal abstraction methods exist, including state abstraction or gradient abstraction. In state abstraction, the values are classified into states, according to given cutoffs that are given by an expert or by data-driven methods, such as equal width discretization (EWD), symbolic aggregate approximation (SAX),[22] or others.[19] Gradient abstraction is applied based on the first derivative, in which the values are classified into gradients of increasing, stable, or decreasing.[19] Eventually, adjacent states, or gradients, having the same values are concatenated into STIs, as illustrated in Figure S1. The output of the temporal abstraction process is a set of ordered STIs, or temporal concepts, in which each STI is defined by a triplet of a start time, an end time, and a symbol.

TIRP mining and temporal relations

TIRPs are defined by a sequence of STIs and a conjunction of the temporal relations among each pair of STIs.[23] Most methods for TIRP mining use a subset of Allen's temporal relations.[24] Allen formulated a finite set of 13 temporal relations between a pair of STIs. The set includes before, meet, overlap, start, contain, finished-by, and their corresponding inverse relations. While Allen's relations were extended by several works, they were mainly for temporal reasoning[20] and not for TIRP discovery, which is the focus of our work. Nevertheless, in the use of TIRPs as features for classification, a broader version was proposed that creates a more generalizing temporal relation that consists of a disjunction of a subset of Allen's relations.[19] The first to use the entire set of Allen's temporal relations were Kam and Fu.[25] However, since

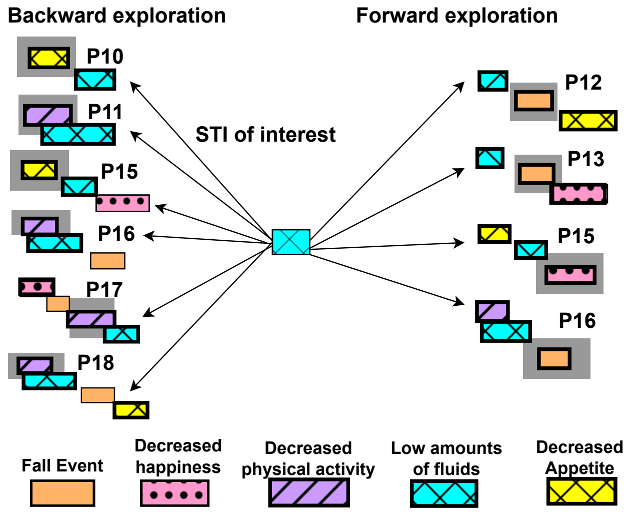

Figure 5. PanTeraV's STI associative exploration example

The STI of interest, <“Low amounts of fluids”>, in the center, is the STI that the user wants to further explore. On the right, all the possible forward extensions of the STI appear, which consist of the STI of interest and at least one additional later STI afterward (in gray background). On the left are all the possible backward extensions.

they did not define the temporal relations among the patterns' components that are not successive, these patterns have been ambiguous. The first to define a non-ambiguous representation of TIRPs based on Allen's relations was Höppner,[26] using a matrix to represent all of the pairwise relations within a TIRP. Initial works introduced naive candidate generation methods.[27,28] The KarmaLego algorithm[23] introduced a direct extension approach, employing the transitivity property of Allen's temporal relations for an efficient candidate generation of TIRP candidates that can exist. Since KarmaLego's index is not quite scalable, an improvement on KarmaLego was proposed, using a more memory-efficient hash-based index. Sharma and Patel[29] introduced the STIPA algorithm as a memory-efficient extension of ARMADA,[30] shrinking its index to fit in devices with strong memory requirements. In Lee et al.,[31] the ZMiner algorithm was proposed, which utilizes a hierarchical lookup hash structure that is used to index the frequent two-sized TIRPs, similar to H-DFS[27] and KarmaLego.[23] In Mordvanyuk et al.,[32] the VertTIRP algorithm was introduced. To accelerate the candidate generation process, VertTIRP uses a pairing strategy that sorts the temporal relations to be assessed, beyond just utilizing the transitivity property as made in KarmaLego. Harel and Moskovitch[17] introduced TIRPClo, the first algorithm for the discovery of all frequent closed TIRPs, using a memory-efficient index and a projection-based technique. Typically, the output of a TIRP mining algorithm is an enumeration tree of all the discovered patterns above the minimal support threshold. Such a tree usually contains hundreds, thousands, or even tens of thousands of patterns (as we use in our user study here), highlighting the necessity of developing methods to efficiently construct and navigate it.

Enumeration tree of TIRPs

The output of a TIRP mining algorithm is an enumeration tree of all discovered frequent TIRPs. Frequent means that the TIRP has a vertical support (VS) above a minimal predefined threshold. “Vertical support” of a TIRP P is defined by the number of transactions (i.e., patient's admissions) in which P holds at least once. Often people refer to relative support, in which the number is divided by the total number of transactions in the database. In addition to the minimal VS threshold, another parameter that is often used in the mining process is the “maximal gap,” which limits the time duration between two STIs in the “before” temporal relation.[23] The value is determined based on what time gap is relevant in the discovery task; however, having a shorter maximal gap will result in less frequent patterns discovered and shorter runtime duration. Thus, having lower minimal VS and a longer maximal gap will result in more frequent patterns. The enumeration tree is organized as follows: the first level of the tree contains all the one-sized TIRPs, which are actually single events or STIs. Below every such TIRP, its extensions appear. For example, in Figure 1, we see that TIRP P6 is an extension of P1, because it contains all the STIs P1 contains and another STI afterward (“Decreased physical activity”). It is possible for a TIRP to have multiple extensions (such as P7). Hence, the k-th level of the tree contains TIRPs in size k, which are the extensions of the previous $k-1$-sized TIRPs that were discovered earlier. As was previously discussed, the input for PanTeraV is the enumeration tree of frequent TIRPs, discovered by a mining algorithm, rather than the original temporal data. Thus, factors that may influence the enumeration tree's size (and hence the input to PanTeraV) are the distribution of the data as well as the parameters for the KarmaLego algorithm.

Except for their frequency, which is measured by their VS, TIRPs have more metrics that were introduced[19] and are used in PanTeraV. “Size” of a TIRP is its number of STIs. “Horizontal support” (HS) of a TIRP P in a transaction is the number of instances of P found in the transaction. Accordingly, the “mean horizontal support” (MHS) of a TIRP P describes the average of HS values of all its supporting transactions. The time duration of a single instance of a TIRP P is defined from its earliest start time of an STI until its last STI's end time. The mean duration (MD) of all the instances of TIRP P in a transaction is defined by the average time duration of each of P's instances in the transaction. Hence, the “mean mean duration” (MMD) of a TIRP P is the average of the MD values over all the supporting transactions. An illustration of the TIRP's metrics can be found in Figure S2.

Visualization of temporal patterns

Most of the works in the past two decades focused mainly on the visualization of a specific temporal pattern and its supporting instances, unlike the focus of our work, which is using visualization to explore an enumeration tree of temporal patterns to identify a specific pattern of interest and its metrics and supporting instances and for associative exploration of STIs. Notable work in temporal pattern visualization systems was introduced by Perer and Wang[16] and Wongsuphasawat and Gotz.[15] Both systems, like similar ones,[12,15,33–36] use Sankey diagrams and alluvial diagrams to visualize the discovered patterns and the connections between the events in them. These diagrams are a type of flow diagram used to visualize changes in data across multiple stages. Mostly the charts are characterized by nodes, representing categories or transactions, connected by bands that show the flow between them. The width of each band is proportional to the average time gap between the nodes, and height

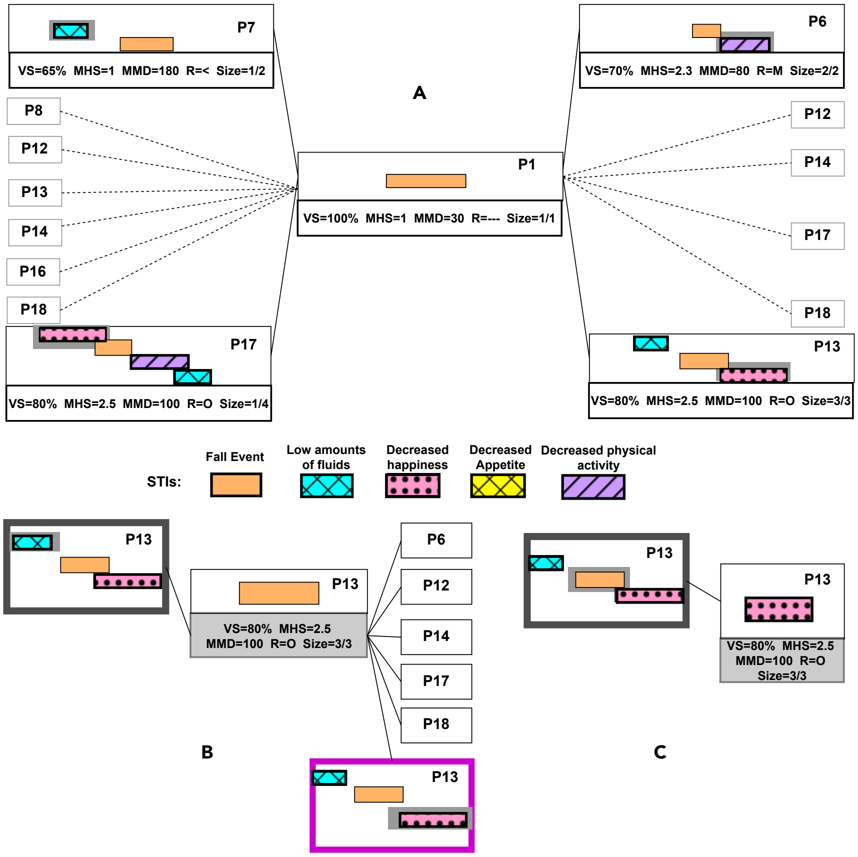

Figure 6. PanTeraV's functionality is demonstrated in a sequence of clicks

(A) The center block represents the <"Fall event"> STI, which is chosen as the STI in focus. Every block from right/left represents extending STIs (with the TIRPs they are part of), similar to Figure 5. The later/earlier STIs to the center STI on the right/left are highlighted in gray background. The metrics of each TIRP are presented at the bottom of the block.

(B) The view after the user selected <"Decreased happiness"> from the right. P13 is marked (purple), and the metrics and label on the center block are updated according to the selected STI. The left side of (B) shows the TIRPs that contain the sequence <"Fall event," "Decreased happiness"> and additional STIs beforehand, thus only P13. P13 on the left is marked in gray to highlight the earlier STI, <"Low amount of fluids">, which was implicitly chosen after the user clicked on P13.

(C) The interface after the user chose to further explore forward in (B), which shifted right. Now, <"Decreased happiness"> is in the center, and the TIRP that associates the earlier STI ("Fall event") is marked (gray) on the left.

is proportional to the number of patients in the transaction. Poon et al.[14] suggested using a 3D sunburst visualization,[13] an advanced variation of the sunburst chart that represents hierarchical data in a circular layout extended into three dimensions. Each event is presented as a colored block, and when a mouse hovers over an event, the visualization highlights the full path to it and the events sequence together with the support values.

While most of the works in the visualization of temporal patterns focused on visualizing a specific pattern and supporting instances, recent studies refer to the problem of visualizing a collection of patterns discovered from a mining process.

KLW, which is the first visualization interface[18] for exploration of the enumeration tree of TIRPs, enables the user to explore the TIRPs using a depth-first strategy in the tree, from the root down the branch through the symbols of the STIs and the temporal relations among them. In each step along the enumeration tree the user can stop and explore a TIRP, view its supporting instances, and explore various metrics and metadata on its supporting transactions, such as their distribution along various demographic properties. The second visualization in KLW enables one to query and filter TIRPs by specifying specific symbols at the beginning, or in the middle, or at the end of the TIRP of interest. KLW can also be used to visualize patterns discovered in a single or in both populations, such as in diabetic and healthy groups, and to facilitate comparisons between patterns identified in the two populations. However, beyond the actual TIRPs, it is of great interest to explore the STIs associatively. For example, looking at a specific symbol, a user would be interested in knowing what typically are the STIs that appear after it, or before, or with any other temporal relation. KLW does not enable the user to move between STIs and explore their relations based on the frequent TIRPs. For example, if the user explores the TIRP P7 (Figure 1), KLW will show only the extensions P12 and P13. The user is not exposed to P18, although it contains the same STIs as P7, because it is not a direct extension of it. PanTeraV presents a new capability that enables one to explore the STIs associatively based on the frequent temporal patterns, allowing the user to navigate both forward and backward and also explore patterns of interest, which we describe in this paper.

METHODS

We introduce PanTeraV, a visual interface that enables the exploration of associative STIs through a collection of frequent temporal patterns structured in an enumeration tree. Although it is demonstrated in the use of TIRPs, it is important to note that the principles introduced in PanTeraV can also be used potentially for other types of temporal patterns, such as sequential patterns[6,37]; semi-interval patterns,[38] which are often used for the analysis and exploration of human interactions[39] but can be applied to any time-stamped data and can be discovered by domain experts or automatically[40]; or other types, including T patterns[41] and spatiotemporal patterns.[42] The input to PanTeraV is the discovered enumeration tree of patterns, irrespective of the algorithm employed for their discovery. We start with formulating the problem, then we introduce the idea of associative exploration and how it can be implemented efficiently by indexing, and last, we introduce PanTeraV and its use.

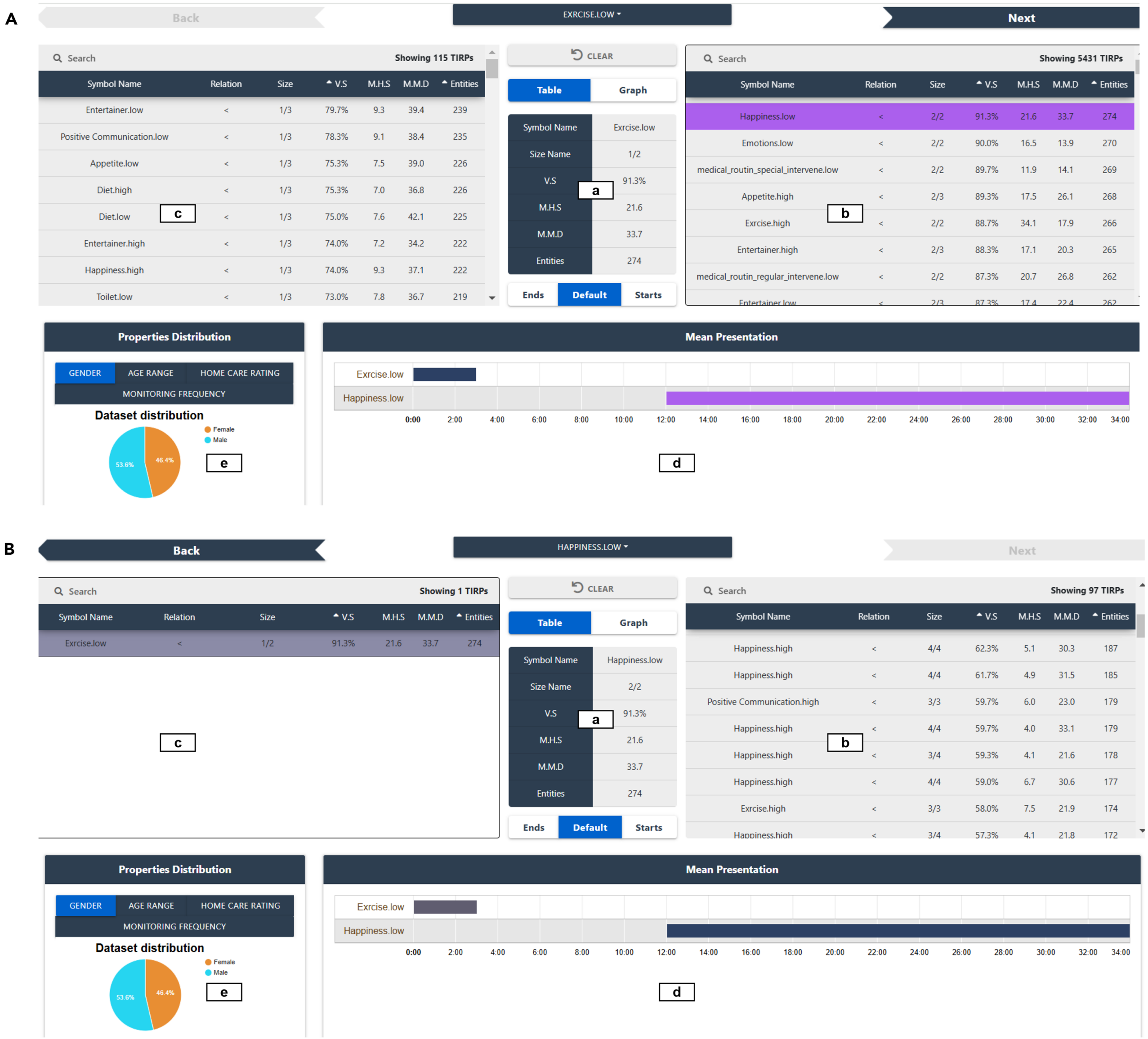

Figure 7. PanTeraV tabular view

Initial search (A) of a specific STI. The user chooses the center STI <"Exercise.Low"> (a) with the metrics of the TIRP that contains only this STI. The right table (b) shows the continuing TIRPs to <"Exercise.Low">, while a specific row with the later STI <"Happiness.High"> is selected (purple). According to the selected row in purple, the left table (c) shows all the preceding TIRPs to the sequence <"Exercise.Low," "Happiness.High">. Timeline visualization (d) shows the explored TIRP's STIs and the temporal relations between them. The pie chart (e) visualizes the demographic distribution of the explored TIRP's supporting transactions (transactions that the TIRP was discovered in) by gender. The next step (B) to (A): the user clicked the "Next" button on the top right corner in (A). This operation shifts the STI sequence. Now, the center STI is <"Happiness.High">. Table (c) shows the previous STI indicating that a forward exploration was done. Table (b) presents all TIRPs that contain additional STIs after the sequence <"Exercise.Low," "Happiness.High">.

Problem formulation

PanTeraV addresses the need to explore STIs through the frequent patterns that were discovered by a mining process. For that purpose, the initial operation that the user is requested to perform is to specify an STI of interest. Given this STI of interest, PanTeraV will show, in any given moment, STIs beforehand or afterward, associated by the frequent patterns in which they appear. For example, Figure 5 demonstrates the associative exploration in PanTeraV. The fixed STI in the center is <"Low amounts of fluids">. The exploration can go either forward (and to extend it into P12, P13, P15, and P16) or backward (to P10, P11, P15, P16, P17, and P18). It is important to mention that forward exploration exposes TIRPs that contain the STI in the center and at least one additional STI afterward.

Similarly, backward exploration presents TIRPs that contain the STI of interest in addition to more STIs beforehand. The exploration is performed dynamically, and when the user wants to choose an STI of interest on the left or on the right, it will be updated accordingly, as will be explained in the next subsection.

CellPress
OPEN ACCESS

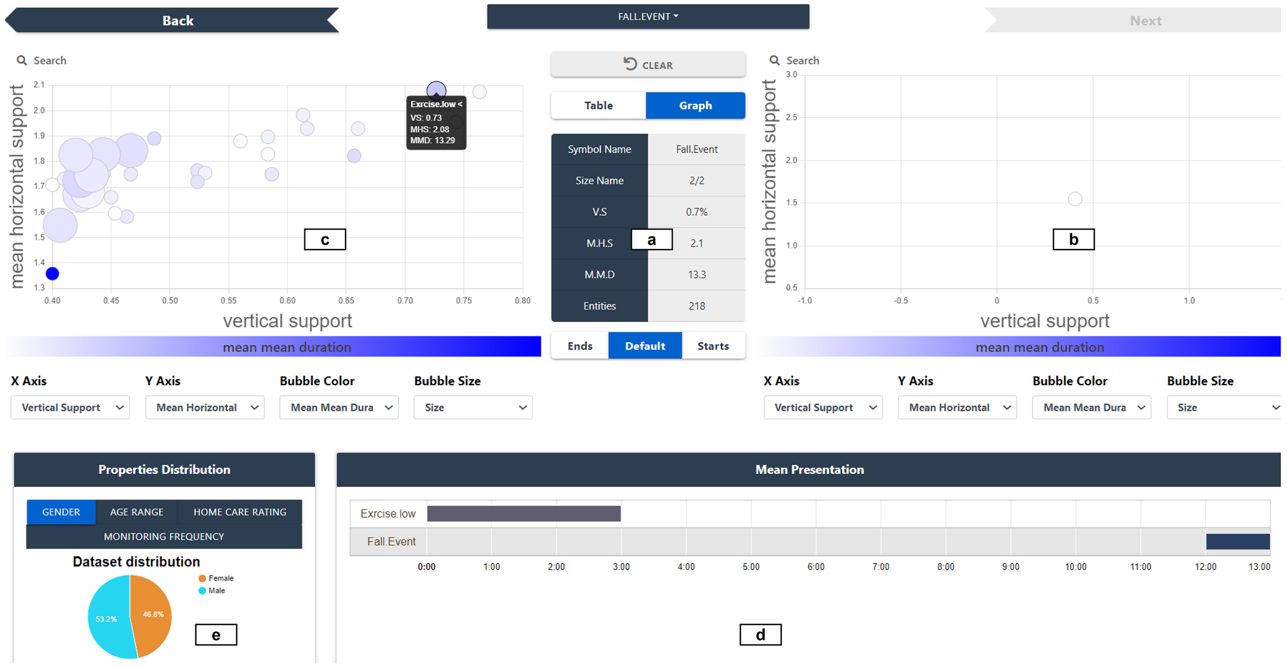

Figure 8. PanTeraV graphical view

Table (a) describes the metrics of the selected TIRP, as was explained in Figure 7. The center STI is <"Fall.Event">. The bubble charts (b) and (c) present later/earlier STIs, respectively, visualized as bubbles. The earlier selected STI "Exercise.Low" is represented using a black line around the bubble. Thus, (b) only presents later STIs to the sequence <"Exercise.Low," "Fall.Event">, which is a single STI. Chart (d) visualizes the selected TIRP, and (e) presents the distribution of the TIRP's supporting transactions by gender.

Indexing for associative exploration

Associative exploration can be either forward or backward, as was demonstrated in the problem formulation section. In order to enable such exploration of the STIs, based on the TIRPs they appear in, we introduce the use of forward and backward indexing. Figure 1 presents a toy example (often there may be hundreds or even thousands of STIs and corresponding TIRPs) of an enumeration tree of TIRPs, which is typically the output of a mining process. Suppose the user explores the TIRP P7, < "Low amounts of fluids" before a "Fall event"> (blue); however, the user wonders what STIs precede or follow the sequence of STIs in P7. Thus, the exploration should expose the user to TIRPs P12, P13, and P16 (purple) or P18 (gray), because they all contain the STIs in P7, in addition to extra STIs beforehand or afterward.

The enumeration tree is constructed in a hierarchical manner and thus does not enable one to move between the mentioned TIRPs directly, because they are not in the same branch. PanTeraV enables associative exploration of all the mentioned TIRPs, using efficient preindexing. Forward indexing builds a mapping of a single STI or a sequence of STIs (TIRP) and all the STIs that continue (forward extend) it. Hence, we can efficiently move from a specific sequence of STIs to its possible extensions (with additional STIs afterward). For example, given the TIRP P7 from Figure 1, the TIRPs that will appear in its forward index are P12, P13, and P18 (purple), because they contain the STIs in P7, in addition to the following STIs ("Decreased appetite" or "Decreased happiness") afterward.

Backward indexing is similar to forward indexing, but the mapping is ordered differently. In the backward indexing, we keep, for a single STI or a sequence of STIs, all the possible backward extensions. For example, for TIRP P7, the TIRPs that appear in its backward index are P16 and P18 (gray) because they consist of the same STIs as P7, in addition to extra STIs beforehand ("Decreased physical activity"). The result of the two indexing techniques is two indices, each consisting of all the STI sequences from the enumeration tree and all their respective extensions based on the common STIs. This approach enables the associative exploration that was demonstrated in the problem formulation section.

Runtime and complexity

It is important to mention that the indexing process occurs only once during PanTeraV initialization with a specific input. The algorithm iterates over the enumeration tree of patterns. For every pattern, the algorithm scans the STIs in it and indexes them twice—for every exploration direction (forward and backward). We denote the number of frequent temporal patterns in the enumeration tree as N, and the largest TIRP (largest number of STIs in a TIRP) as $Lmax$. Thus, the runtime complexity of the indexing algorithm is $O(N) * O(Lmax)$. Note that $Lmax$ is typically half a dozen or about a dozen STIs, while N is often hundreds or even tens of thousands of TIRPs, as in our user study.

The resulting data structures from the indexing algorithm are two nested hashmaps (forward and backward). Every hashmap contains, for every pair of STIs (ordered by their occurrence in the TIRP), all the TIRPs that contain them, one after the other, with their temporal relations. For example, in the forward

hashmap of <"Low amount of fluids">, the symbol < "Fall event"> will appear with the connected TIRPs <P7, P12, P13, P18>, because all these TIRPs contain both STIs < "Low amount of fluids"> and <"Fall event">, each with its temporal relation between the STIs. We denote the number of symbols in the dataset as M. Hence, the space complexity is $O(M) * O(M) * O(N)$.

PanTeraV's functionality

To describe the functionality of PanTeraV and how to move between the STIs, we will use the following terms: "earlier STI" is the immediate STI before the STI in focus in a specific TIRP. For example, <"Low amounts of fluids"> is the earlier STI to < "Fall event"> in TIRP P13. The first operation that the user is required to do in order to start exploring using PanTeraV is to choose a single STI to focus on. Afterward, PanTeraV will present all the extending STIs (with their associated TIRPs) from the forward and backward indices. "Later STI" is the immediate STI after the STI in focus in a specific TIRP. For example, < "Decreased happiness"> is the later STI to <"Fall event"> in TIRP P13 (Figure 5).

Figure 6A demonstrates the selection of <"Fall event"> as the initial STI. Thus, the center block presents P1—the one-sized TIRP, which contains this STI. On the sides, all the later and earlier STIs to <"Fall event"> appear together with their associated TIRPs, as they appear in the indices. The later/earlier STIs to the STI in focus are marked using gray background. Note that a TIRP can appear on both sides if it contains both earlier and later STIs to the center STI (such as P12, P13, P14, P17, and P18). Among the metrics that are shown below every block, VS, MHS, and MMD are as defined in the background section. R represents the temporal relation between the center STI and the earlier/later STI on the sides. For example, P17 on the left in Figure 6 presents R = O because <"Decreased happiness"> (earlier STI) overlaps (O) with the center STI ("Fall event") in P17. Size is presented as X/Y, in which Y is the size (number of STIs) of the TIRP and X is the location of the earlier/later STI in the TIRP. For example, the size of P17 is $1/4$ because P17 contains four STIs (Y) and <"Decreased happiness"> (earlier STI) is the first in the TIRP.

Focused exploration

The user can explore the STIs by clicking on a specific block (which presents a later/earlier STI) on the sides. Figure 6B shows PanTeraV after selecting <"Decreased happiness"> on the right from Figure 6A. After the selection, P13 is marked (purple) in Figure 6B to indicate that the user selected a later STI. In addition, this selection fixed the sequence of STIs <"Fall event," "Decreased happiness">. Hence, the presented STIs on the left in Figure 6B are STIs in TIRPs that contain the sequence < "Fall event," "Decreased happiness"> and at least one additional STI beforehand. The only TIRP that satisfies this condition is P13, with the additional <"Low amounts of fluids">. Due to the fact that the metrics in the center block always update according to the selected blocks on the sides, after selecting P13, the metrics represent it. The size in the center block is now 2/3 because the center STI <"Fall event"> is the second STI out of three STIs in P13.

Further forward/backward exploration

After the user selects a later/earlier STI, further exploration in this direction is enabled. Figure 6C demonstrates forward exploration. Similarly, given a selected earlier STI, backward exploration is enabled. It is important to mention that further exploration does not change the selected sequence of STIs. Thus, after the shift right (from Figure 6B to 6C), the metrics on the center block still represent P13 (the change is the visualized STI in the center). The earlier STI on the left in Figure 6C is <"Fall event"> with the associated TIRP P13. P13 is marked (gray) to indicate that an earlier STI was chosen. There are no later STIs in Figure 6C because there are no TIRPs that contain the sequence <"Fall event," "Decreased happiness"> and another later STI.

Note: further exploration can go on and allow the user to discover much larger TIRPs and not only those that consist of the earlier, center, and later STIs. Every shift (whether forward or backward) exposes more STIs that can be further extended. The selected STIs are fixed as a sequence, and with every shift the user can add more STIs to the sequence.

PanTeraV interfaces

PanTeraV consists of two different visual interfaces, each with its own advantages. The first interface is the tabular view. The main purpose of the tabular view is to enable efficient exploration of STIs by choosing to extend a sequence of STIs with an earlier/later STI as was demonstrated in the previous section. The possible extensions for the selected STI sequence are presented using dynamic tables. Every row in a table represents an extending STI (with its associated TIRP). The graphical view enables exploration in a different way. The graphical view presents the possible extensions using bubble charts, where every bubble on the chart represents an earlier/later STI with its associated TIRP. The axes of the chart are the metrics of the TIRP. Both interfaces are enabled in the system, and the user can easily move between them by clicking a button.

PanTeraV—Tabular view

PanTeraV's tabular interface's main purpose is to allow the user to explore the STIs' associations by selecting later/earlier STIs that are presented on the right/left as illustrated in Figure 6. Figure 7 presents the tabular view of PanTeraV and contains two parts, Figure 7A followed by Figure 7B, demonstrating two steps in the exploration process, as we describe here. In each of the parts of the figure, subregions exist: (a) presents the center STI, which is now the focus of the user. After the user selects a specific extending STI (i.e., row) from one of the tables, the counter table will update accordingly.

Figure 7A (a) demonstrates PanTeraV after the user selected "Happiness.High" from table (b), marked in purple as the later STI; thus, table (c) shows only the earlier STIs to the sequence <"Exercise.Low," "Happiness.High">. This is similar to the transition in Figure 6 from (a) to (b). Note that all TIRPs on the left (c) are at least in size 3 because they contain the selected sequence (<"Exercise.Low," "Happiness.High">) and at least one additional earlier STI.

The explored TIRP is visualized at the bottom using a timeline chart, Figure 7A (d), with its mean presentation, which includes the STIs' MD, based on the supporting instances. Each of the TIRP's STIs is presented in a different row and a different color on the timeline. The dark blue STI corresponds to the center STI. If there is a selected later STI from table (b), a purple STI that corresponds to it will be shown as well (as demonstrated in a). Similarly, if an earlier STI is selected from table (c), a gray

STI will be shown. Information about the demographic properties of the explored TIRP's supporting transactions is visualized using a pie chart (e). For example, (e) shows how the supporting transactions are distributed by gender, for which, in this case, the proportions are almost the same. Further exploration is enabled given that the user selected a later/earlier STI from table (b) or (c), respectively.

Figure 7B demonstrates this case. The user shifted-right (further explored forward), and now the new center STI is "Happiness.High" and the earlier STI (gray) is "Exercise.Low" (which was the center STI in Figure 7A). Note that the metrics in table (a) remained unchanged (except size) because the explored TIRP is the same, the only change is the STI in the center. Table (b) was updated and now shows the later STIs to the sequence <"Exercise.Low," "Happiness.High">. Since the explored TIRP was not changed, the pie chart (e) of its supporting transactions shows the same distribution. Moreover, the visualized TIRP in the timeline chart (d) presents the same STIs, except for a change in their colors—while the STI in the center is always blue.

PanTeraV—Graphical view

The graphical view shows the same information as the tabular view, but in a way that provides a fast idea of the properties of the patterns. It intends to provide an overview of the later/earlier STIs using a bubble chart. The bubble chart shows the earlier/later STIs as bubbles, and the axes of the chart represent the associated TIRPs' metrics, such as the size and color.

It is important to mention that the presented metrics are temporal metrics, which distinguish one TIRP from another. These metrics were discussed in detail in the background section. TIRP metadata, such as static properties (age, sex, etc.), do not influence the difference between TIRPs, hence are not shown in the bubble chart but in the pie chart (e).

Every bubble on the bubble chart corresponds to a row in a table (Figure 7). The graph's axes can be changed and swapped, which will result in a different arrangement of the bubbles on the graph. Figure 8 presents the PanTeraV graphical view. Table (a) presents the STI in focus, together with the metrics of the selected TIRP as was explained regarding the tabular view. The bubble charts (b) and (c) correspond to tables (b) and (c) in Figure 7 and present later/earlier STIs, associated with their TIRPs that are visualized as bubbles. Every bubble in the graphical view is located on the chart according to the associated TIRP's metrics values. For example, the selected bubble on the left (chart [c]) is located on $(0.73, 2.08)$ because its VS (x axis) is 73%, and the MHS (y axis) is 2.08. The bubble's color tone is set according to the TIRP's MMD (which is 13.29). The higher the TIRP's MMD, the darker the bubble's color (according to the scale below the graph). Note that (b) presents only one bubble because after selecting the earlier STI (f), which is "Food.Low", later STIs in (b) are extensions to the sequence <"Exercise.Low," "Fall.Event">, which results in only a single TIRP.

The graphical view, similar to the tabular view, enables further exploration. After the user selects a bubble from the side graphs, further exploration in this direction is enabled. For example, after the user selects the earlier STI with its TIRP (f), the interface enables one to click on the "Back" button on the top left corner. The main advantage of the PanTeraV's graphical interface is that the bubble chart enables the user to derive fast conclusions about the TIRPs associated with the later/earlier STIs. For example, if the user is interested in identifying the most frequent preceding TIRPs of "Fall.Event," where the x axis on the bubble chart represents the VS, the user needs to locate the rightmost TIRPs, which is faster than manually searching for these TIRPs in a table.

Experimental procedures

User study

We conducted a user study to evaluate the usability of PanTeraV. Specifically, our goal was to assess whether users can effectively utilize both forward and backward exploration in PanTeraV and whether users without prior knowledge of temporal mining can be equally effective in using these features. Users were asked to conduct a series of tasks and were evaluated on the accuracy of their answers and the time it took them to complete the tasks. KLW was selected as the baseline interface for comparison since it is the only interface in the literature that facilitates the exploration of TIRPs from an enumeration tree of frequent temporal patterns.

The metrics for user study evaluation were time duration and accuracy, to objectively evaluate the results and because those metrics also were used to evaluate the baseline interface (KLW). Accuracy was considered because each question had only one correct answer, which was predetermined and written ahead of time to ensure fairness. The time duration was measured only after the subject finished reading the question, understood it, and began interacting with the interface. In this way, we avoided bias related to the time it took to read the question or understand the instructions, which was not part of the evaluation.

We recruited 15 volunteers to participate in the user study, which followed a protocol approved by the departmental ethics committee (no. SISE-2022-28). No compensation was offered in exchange for participation and participants could leave the study at any point. All participants provided their informed consent. Participants were undergraduate and graduate students in our department with basic training in data science. Eight participants had additional experience in temporal mining (TM group) either from taking a class on the subject or from using temporal mining methods, and the remaining seven participants had no more than basic training in data science (DS group). The study began with a short, 15-min introduction to temporal pattern mining and the usage of the PanTeraV interface as well as the KLW baseline interface. Then, participants were asked to complete a series of tasks using the web interfaces while their answers were recorded by the experimenter. The study took participants 30-45 min to complete.

Participants were asked to answer three types of questions using the different interfaces. First, there were basic usability questions that tested participants' basic understanding of the interface. A second type of question, which is the key focus of the study, involved backward exploration questions. This type of question required participants to identify patterns that precede a single event or a sequence of events. Since answering this kind of question is much harder with existing interfaces, we allowed participants to skip the question if they thought answering it would require more than 3 min or if they have not provided an answer after that allotted time. Finally, a third type of question evaluated participants' ability to use forward

exploration, mostly as a robustness check to ensure that this important functionality can still be efficiently used and not hampered by the additional functionality. Participants were asked to complete the tasks using the different interfaces (two interfaces of PanTeraV and two interfaces of KLW) on two different datasets as described next. Each participant answered the questions on all eight combinations of interface and dataset, with the order of these combinations being randomized and counter-balanced across participants. The full set of questions can be found in Data S1.

To thoroughly test usability we used two real-world datasets from different domains. The first was the Diabetes dataset, provided through a collaboration with Clalit Health Services and containing 835,168 observations on 2,004 chronic type 2 diabetes patients. The dataset contains six temporal variables (laboratory values or interventions) recorded over time for each patient.[19] Each patient was described by 5 years of data in monthly granularity, which were abstracted using the gradient abstraction method.[19] To discover frequent TIRPs, the KarmaLego algorithm was applied to the data with minimal VS of 20% and maximal gap value of 20 months, which resulted in 3,262 frequent patterns.

The second dataset was the Falls dataset, which contains 1,048,576 observations from over 1,700 care homes across the United Kingdom. The data span 3 years (2017–2019) and contain routinely collected information about care home residents using the Mobile Care Monitoring app developed by Person Centred Software.[43] The dataset contains 78 temporal variables (daily routine values or interventions) recorded over time for each resident. Residents' temporal variables were abstracted using equal width discretization, and KarmaLego was used with minimal VS of 40% and a max gap of 20 days, which resulted in 61,168 frequent patterns. We used higher minimal support in the Falls dataset due to its larger size and the corresponding size of the discovered TIRP set.

The datasets for the user study were chosen for several reasons. First, they were selected to experiment with a variety of types of temporal data. While the Diabetes dataset includes regularly (monthly) sampled data, including clinical data, such as glucose levels in the blood or LDL cholesterol measures, the Falls dataset consists of daily irregularly logged data, including continuous or discrete values, such as drinking coffee and eating. Thus, while the Diabetes dataset shows that we work with a dataset in which all temporal variables are uniform and have values in each time stamp, the Falls dataset shows the use of a dataset that is sparse, having irregularly logged data of the various temporal variables. We also wanted to use different domains of data, although PanTeraV is generic and is not limited to specific domains of data. However, the purpose of PanTeraV is to enable exploration of forward and backward associations of TIRPs and STIs through visualization. The use of temporal abstraction enables us to work with heterogeneous multivariate temporal data.

RESOURCE AVAILABILITY

Lead contact

Requests for further information and resources should be directed to and will be fulfilled by the lead contact, Tali Malenboim (talimal@post.bgu.ac.il).

Materials availability

All code for the PanTeraV system can be found in the OSF repository https://doi.org/10.17605/OSF.IO/UAFV9.[44] The presented dataset in the repository is the Diabetes dataset, which can be integrated into the system to explore the discovered TIRPs. The Falls dataset is not provided in the mentioned repository due to confidentiality reasons. Interested readers can contact talimal@post.bgu.ac.il for further information.

Data and code availability

All original code has been deposited at https://github.com/Talimal/PanTeraV_backend and https://github.com/Talimal/PanTeraV_frontend and is publicly available at https://doi.org/10.17605/OSF.IO/UAFV9 as of the date of publication.

ACKNOWLEDGMENTS

T.M. was funded by the Kreitman School of Advanced Graduate Studies and Israeli Ministry of Science and Technology grant 1001577517 and British Council BIRAX 5531.

AUTHOR CONTRIBUTIONS

Conceptualization, T.M. and R.M.; methodology, T.M. and R.M.; software, T.M.; validation, T.M. and N.G.; formal analysis, T.M. and N.G.; investigation, T.M.; data curation, T.M.; writing – original draft, T.M., R.M., and N.G.; writing – review & editing, T.M., R.M., and N.G.; visualization, T.M.; supervision, R.M. and N.G.; project administration, R.M. and N.G.; funding acquisition, R.M.

DECLARATION OF INTERESTS

The authors declare no competing interests.

SUPPLEMENTAL INFORMATION

Supplemental information can be found online at https://doi.org/10.1016/j.patter.2025.101292.

Received: August 19, 2024
Revised: March 8, 2025
Accepted: May 16, 2025
Published: June 11, 2025

REFERENCES

1. Combi, C., and Shahar, Y. (1997). Temporal reasoning and temporal data maintenance in medicine: issues and challenges. Comput. Biol. Med. *27*, 353–368. https://doi.org/10.1016/s0010-4825(96)00010-8.
2. Bellazzi, R., Ferrazzi, F., and Sacchi, L. (2011). Predictive data mining in clinical medicine: a focus on selected methods and applications. WIREs Data Min. &. Knowl. *1*, 416–430. https://doi.org/10.1002/widm.23.
3. Moskovitch, R. (2022). Multivariate temporal data analysis—a review. WIREs Data Min. &. Knowl. *12*, e1430. https://doi.org/10.1002/widm.1430.
4. Moskovitch, R., Polubriaginof, F., Weiss, A., Ryan, P., and Tatonetti, N. (2017). Procedure prediction from symbolic electronic health records via time intervals analytics. J. Biomed. Inform. *75*, 70–82. https://doi.org/10.1016/j.jbi.2017.07.018.
5. Moskovitch, R., Walsh, C., Wang, F., Hripcsak, G., and Tatonetti, N. (2015). Outcomes prediction via time intervals related patterns. In 2015 IEEE international conference on data mining. IEEE, pp. 919–924. https://doi.org/10.1109/icdm.2015.143.
6. Han, J., Pei, J., Mortazavi-Asl, B., Pinto, H., Chen, Q., Dayal, U., and Hsu, M. (2001). Prefixspan: Mining sequential patterns efficiently by prefix-projected pattern growth. In proceedings of the 17th international conference on data engineering (Citeseer), pp. 215–224. https://doi.org/10.1109/icde.2001.914830.

7. Harel, O.D., and Moskovitch, R. (2021). Complete closed time intervals-related patterns mining. Proc. AAAI Conf. Artif. Intell. *35*, 4098–4105. https://doi.org/10.1609/aaai.v35i5.16531.
8. Goren-Bar, D., Shahar, Y., Galperin-Aizenberg, M., Boaz, D., and Tahan, G. (2004). Knave ii: the definition and implementation of an intelligent tool for visualization and exploration of time-oriented clinical data. In Proceedings of the working conference on Advanced visual interfaces, pp. 171–174. https://doi.org/10.1145/989863.989889.
9. Klimov, D., Shahar, Y., and Taieb-Maimon, M. (2010). Intelligent visualization and exploration of time-oriented data of multiple patients. Artif. Intell. Med. *49*, 11–31. https://doi.org/10.1016/j.artmed.2010.02.001.
10. Shahar, Y., Goren-Bar, D., Galperin, M., Boaz, D., and Tahan, G. (2003). Knave-ii: A distributed architecture for interactive visualization and intelligent exploration of time-oriented clinical data. In Proceedings of Intelligent Data Analysis in Medicine and Pharmacology, Protaras, Cyprus. https://doi.org/10.1016/j.artmed.2005.03.001.
11. Gotz, D., Wang, F., and Perer, A. (2014). A methodology for interactive mining and visual analysis of clinical event patterns using electronic health record data. J. Biomed. Inform. *48*, 148–159. https://doi.org/10.1016/j.jbi.2014.01.007.
12. Gotz, D., and Stavropoulos, H. (2014). Decisionflow: Visual analytics for high-dimensional temporal event sequence data. IEEE Trans. Vis. Comput. Graph. *20*, 1783–1792. https://doi.org/10.1109/tvcg.2014.2346682.
13. Rodden, K. (2014). Applying a sunburst visualization to summarize user navigation sequences. IEEE Comput. Graph. Appl. *34*, 36–40. https://doi.org/10.1109/mcg.2014.63.
14. Poon, L.K., Kong, S.-C., Yau, T.S., Wong, M., and Ling, M.H. (2017). Learning analytics for monitoring students participation online: Visualizing navigational patterns on learning management system. In International conference on blended learning (Springer), pp. 166–176. https://doi.org/10.1007/978-3-319-59360-9_15.
15. Wongsuphasawat, K., and Gotz, D. (2011). Outflow: Visualizing patient flow by symptoms and outcome. In IEEE VisWeek Workshop on Visual Analytics in Healthcare, Providence, Rhode Island, USA (American Medical Informatics Association), pp. 25–28.
16. Perer, A., and Wang, F. (2014). Frequence: Interactive mining and visualization of temporal frequent event sequences. In Proceedings of the 19th international conference on Intelligent User Interfaces, pp. 153–162. https://doi.org/10.1145/2557500.2557508.
17. Harel, O., and Moskovitch, R. (2023). Tirpclo: efficient and complete mining of time intervals-related patterns. Data Min. Knowl. Discov. *37*, 1806–1857. https://doi.org/10.1007/s10618-023-00944-6.
18. Shitrit, G., Tractinsky, N., and Moskovitch, R. (2022). Visualization of frequent temporal patterns in single or two populations. J. Biomed. Inform. *134*, 104169. https://doi.org/10.1016/j.jbi.2022.104169.
19. Moskovitch, R., and Shahar, Y. (2015). Classification of multivariate time series via temporal abstraction and time intervals mining. Knowl. Inf. Syst. *45*, 35–74. https://doi.org/10.1007/s10115-014-0784-5.
20. Freksa, C. (1992). Temporal reasoning based on semi-intervals. Artif. Intell. *54*, 199–227. https://doi.org/10.1016/0004-3702(92)90090-k.
21. Miller, C., and Quek, F. (2011). Toward multimodal situated analysis. In Proceedings of the 13th international conference on multimodal interfaces, pp. 239–246. https://doi.org/10.1145/2070481.2070526.
22. Lin, J., Keogh, E., Lonardi, S., and Chiu, B. (2003). A symbolic representation of time series, with implications for streaming algorithms. In Proceedings of the 8th ACM SIGMOD workshop on Research issues in data mining and knowledge discovery, pp. 2–11. https://doi.org/10.1145/882082.882086.
23. Moskovitch, R., and Shahar, Y. (2015). Fast time intervals mining using the transitivity of temporal relations. Knowl. Inf. Syst. *42*, 21–48. https://doi.org/10.1007/s10115-013-0707-x.
24. Allen, J.F. (1990). Maintaining knowledge about temporal intervals. Commun. ACM *26*, 361–372. https://doi.org/10.1016/b978-1-4832-1447-4.50033-x.
25. Kam, P.-s., and Fu, A.W.-C. (2000). Discovering temporal patterns for interval-based events. In International Conference on Data Warehousing and Knowledge discovery (Springer), pp. 317–326. https://doi.org/10.1007/3-540-44466-1_32.
26. Höppner, F. (2001). Learning temporal rules from state sequences. IJCAI Workshop on Learning from Temporal and Spatial Data, *25* (Citeseer).
27. Papapetrou, P., Kollios, G., Sclaroff, S., and Gunopulos, D. (2009). Mining frequent arrangements of temporal intervals. Knowl. Inf. Syst. *21*, 133–171. https://doi.org/10.1007/s10115-009-0196-0.
28. Patel, D., Hsu, W., and Lee, M.L. (2008). Mining relationships among interval-based events for classification. In Proceedings of the 2008 ACM SIGMOD international conference on Management of data, pp. 393–404. https://doi.org/10.1145/1376616.1376658.
29. Sharma, A.K., and Patel, D. (2018). Stipa: A memory efficient technique for interval pattern discovery. In 2018 IEEE International Conference on Big Data (Big Data) (IEEE), pp. 1767–1776. https://doi.org/10.1109/bigdata.2018.8622421.
30. Winarko, E., and Roddick, J.F. (2007). Armada–an algorithm for discovering richer relative temporal association rules from interval-based data. Data Knowl. Eng. *63*, 76–90. https://doi.org/10.1016/j.datak.2006.10.009.
31. Lee, Z., Lindgren, T., and Papapetrou, P. (2020). Z-miner: an efficient method for mining frequent arrangements of event intervals. In Proceedings of the 26th ACM SIGKDD International Conference on Knowledge Discovery & Data Mining, pp. 524–534. https://doi.org/10.1145/3394486.3403095.
32. Mordvanyuk, N., López, B., and Bifet, A. (2021). verttirp: Robust and efficient vertical frequent time interval-related pattern mining. Expert Syst. Appl. *168*, 114276. https://doi.org/10.1016/j.eswa.2020.114276.
33. Froehlich, P. (2005). Interactive sankey diagrams. In IEEE Symp. On Information Visualization, *233*. https://doi.org/10.1109/infvis.2005.1532152.
34. Wongsuphasawat, K., Guerra Gómez, J.A., Plaisant, C., Wang, T.D., Taieb-Maimon, M., and Shneiderman, B. (2011). Lifeflow: visualizing an overview of event sequences. In Proceedings of the SIGCHI conference on human factors in computing systems, pp. 1747–1756. https://doi.org/10.1145/1979742.1979557.
35. Guo, Y., Guo, S., Jin, Z., Kaul, S., Gotz, D., and Cao, N. (2022). A survey on visual analysis of event sequence data. IEEE Trans. Vis. Comput. Graph. *28*, 5091–5112. https://doi.org/10.1109/tvcg.2021.3100413.
36. Monroe, M., Meyer, T.E., Plaisant, C., Lan, R., Wongsuphasawat, K., Coster, T.S., Gold, S., Millstein, J., and Shneiderman, B. (2013). Visualizing patterns of drug prescriptions with eventflow: A pilot study of asthma medications in the military health system. Tech. Rep. OFFICE OF THE SURGEON GENERAL (ARMY) FALLS CHURCH VA. https://doi.org/10.21236/ada588017.
37. Zaki, M.J. (2001). Spade: An efficient algorithm for mining frequent sequences. Mach. Learn. *42*, 31–60. https://doi.org/10.1023/A:1007652502315.
38. Mörchen, F., and Fradkin, D. (2010). Robust mining of time intervals with semi-interval partial order patterns. In Proceedings of the 2010 SIAM international conference on data mining (SIAM), pp. 315–326. https://doi.org/10.1137/1.9781611972801.28.
39. Miller, C., and Quek, F. (2012). Interactive data-driven discovery of temporal behavior models from events in media streams. In Proceedings of the 20th ACM international conference on Multimedia, pp. 459–468. https://doi.org/10.1145/2393347.2393413.
40. Miller, C., and Miller, C. (2019). Timing is everything: Identifying diverse interaction dynamics in scenario and non-scenario meetings. In 2019 15th International Conference on eScience (eScience) (IEEE), pp. 203–212. https://doi.org/10.1109/escience.2019.00029.

41. Magnusson, M.S. (2019). T-pattern detection and analysis (tpa) with themetm: a mixed methods approach. Front. Psychol. *10*, 2663. https://doi.org/10.3389/fpsyg.2019.02663.

42. Chang, A.Y., and Yu-Wen, C. (2018). Dynamic data representations for spatio-temporal data visualization. In Frontier Computing: Theory, Technologies and Applications FC 2016 5 (Springer), pp. 667–675. https://doi.org/10.1007/978-981-10-3187-8_63.

43. Dvir, O., Wolfson, P., Lovat, L., and Moskovitch, R. (2020). Falls prediction in care homes using mobile app data collection. In International Conference on Artificial Intelligence in Medicine (Springer), pp. 403–413. https://doi.org/10.1007/978-3-030-59137-3_36.

44. Moskovitch, T. (2025). Panterav (OSF). https://doi.org/10.17605/OSF.IO/UAFV9.

Patterns, Volume *6*

Supplemental information

Visualization of associative exploration of temporal concepts via frequent patterns

Tali Malenboim, Nir Grinberg, and Robert Moskovitch

Figure S1. Temporal Abstraction

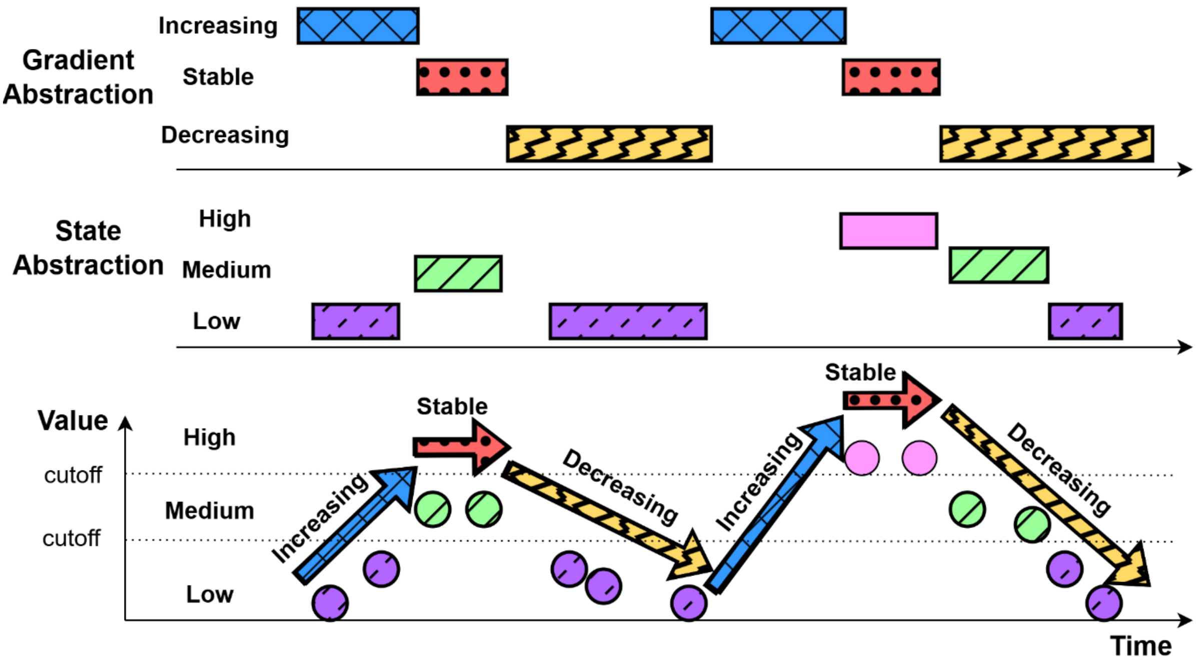

Raw time point values (at the bottom) are abstracted into symbolic time intervals series using State Abstraction (at the middle) with three state symbols (Low, Medium, High) and using Gradient Abstraction (at the top) with three gradient symbols (Increasing, Stable, Decreasing). The symbolic time intervals in the state abstraction chart correspond to the colored time point values (according to their value) in the bottom chart. The colored symbolic time intervals in the gradient abstraction chart correspond to the arrows that represent the gradients at the bottom chart.

Supplemental Note S1.

The process of transforming raw heterogeneous temporal data, such as time series, into symbolic time point series is called temporal abstraction (TA). Temporal abstraction encompasses various types, including state abstraction, gradient abstraction, SAX (Symbolic Aggregate approXimation), and more.

State Abstraction categorizes every time point value from the raw data to a state according to predefined cutoffs (such as low, medium or high glucose levels in blood tests), and later adjacent states having the same value are being concatenated into a symbolic time interval. Gradient Abstraction calculates the gradient of k recent time point values and concatenates the adjacent time points into a single STI which is labeled with the gradient (decreasing, stable or increasing).

For example, figure S1 demonstrates the process of two different TA methods on the same dataset. The chart at the bottom presents a univariate time series. The middle chart presents the state abstraction, applied on the dataset.

As can be seen, the first two time point values are categorized into the low state (since they are below the first cutoff), which is colored in purple. Since both values are in low state, they are concatenated into a symbolic time interval, which appears first in the (intermediate) state abstraction chart. Thus, the STIs in the intermediate chart are the state abstraction of the raw time point series at the bottom.

In the same manner, the top chart presents the result of applying the gradient abstraction of time point values at the bottom chart. The first blue arrow in the bottom chart describes the gradient of the first time point values, which is increasing, followed by a stable gradient colored in red. Looking at the top chart there are the corresponding STIs, having the first colored with blue for the increasing arrow, followed by the stable STI colored red, and so on.

Figure S2. TIRPs metrics example among three transactions

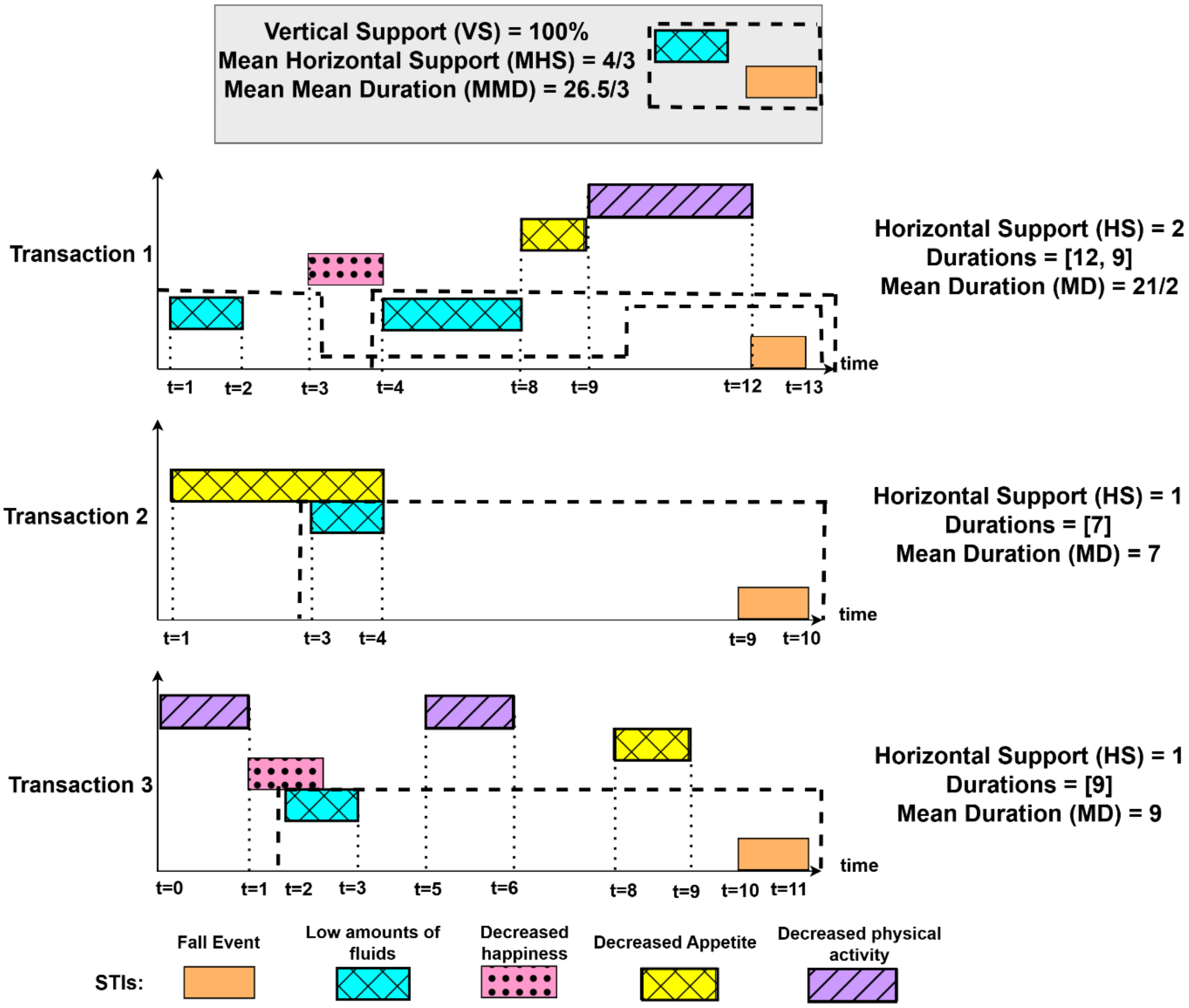

The explored TIRP is <'Low amounts of fluids, before a Fall Event'>, as illustrated in the gray rectangle in the top of the figure. The text on the right of every timeline, presents the TIRP's metrics in every supporting transaction. The overall TIRP's metrics are presented in the gray rectangle at the top.

Supplemental Note S2.

The output of a TIRPs mining algorithm is a set of all discovered frequent TIRPs.

TIRPs are commonly characterized by a number of metrics, that were explained briefly in the background section. Figure S2 shows an example of TIRP <'Low amounts of fluids, before a Fall Event'> and its metrics.

Every timeline represents the occurrences (instances) of the TIRP among its supporting transactions (transactions which the TIRP was discovered in). In addition to the TIRP's STIs, there are more STIs in the transactions, that are not part of this specific TIRP (such as 'Decreased Appetite'). The text on the right of every transaction's graph, presents the metrics of the TIRP in this specific transaction. For example, transaction 1, has two instances of <'Low amounts of fluids, before a Fall Event'> in the data, thus the Horizontal Support (HS) of this TIRP in transaction 1 is two. In contrast, the HS of this TIRP in transaction 2 and transaction 3 is one, only one single instance of this TIRP in these transactions. Thus, the Mean Horizontal Support (MHS) of this TIRP is the average of all the HS among the supporting transactions, which is 4/3.

The Vertical Support (VS) of this TIRP is 100%, because it appears in all the transactions in the dataset (given only these three transactions).

The Duration of a TIRP is set according to a specific instance of this TIRP in a specific transaction. For example, transaction 1 has two instances of TIRP <'Low amounts of fluids, before a Fall Event'> in the data, thus there are two durations in this transaction, which are 12 and 9. The first one starts in t=1 and ends with t=13, and the second starts in t=4 and ends with t=13. the Mean Duration (MD) in this transaction is the average of the TIRP's instances durations, which is 10.5. The Mean Mean Duration (MMD) is calculated as the average of all MDs of the TIRP, among all transactions, which is 26.5/3 in this example.

Figure S3, related to figure 7. Percentage of participants to answer exploration questions (y-axis) in a specific time range (x-axis).

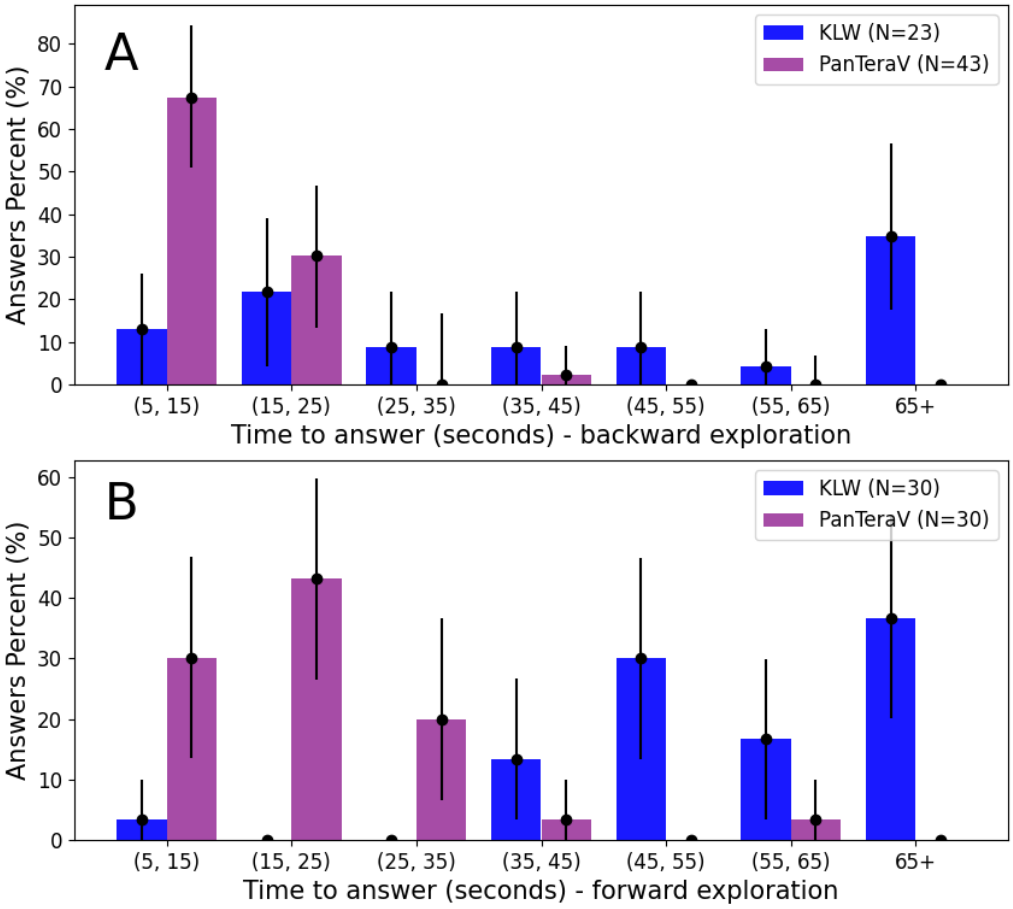

Panel A shows the results for backwards exploration questions, panel B for forward questions.

Supplemental Note S3.

In addition to figure 7, we show the results without cumulative calculation over time.

Figure S3 shows the percentage of correct answers versus the time it took participants to get them, both for backward (panel A) and forward (panel B) exploration.

As was mentioned in the results section, the number of answers reported is the number of participants that completed the tasks, after more than 5 seconds.

In about 35% of cases, participants who diligently attempted to answer the questions using the KLW interface,spent over 65 seconds on the task and often the full 90 seconds.

Yet, none of them were able to obtain the correct answer using the KLW interface.

In contrast, participants using the PanTeraV interface answered all of the questions correctly, and as shown in the figure, most of them took 5-15 seconds to get to the answer (67%) or between 15 to 25 seconds (30%).

Panel B of figure S3 shows the time it took participants to complete forward exploration tasks.

One can see that the vast majority of participants (a total of 95%) needed more than 35 seconds to complete the task using the KLW interface.

Using the same set of forward exploration questions, Panel B shows that most participants (~ 93%) using the PanTeraV interface took less than 35 seconds to complete the task.

In terms of accuracy, participants answered correctly at a high rate using both interfaces (98% for PanTeraV and 91% KLW interfaces) and those rates were not statistically different (P=0.17).

In summary, the results show that PanTeraV enables participants to tackle the same set of exploration questions significantly faster than using the KLW interface.

Data S1: user study questions divided to basic/exploration questions, per dataset and interface

Basic Questions - Diabetes Dataset

KLW Tabular baseline

What type of event reoccurs the most in the dataset (mean horizontal support)?

KLW Search baseline

Which age group is more likely to experience a period of an increase in HBA1C?

PanTeraV Tabular

What are the types of events that occur the most ACROSS patients (vertical support of at least 99)?

PanTeraV Graphical}

Which age group is more likely to experience a period of an increase in LDL?

Basic Questions - Falls Dataset

KLW Tabular baseline

What are the two types of events that reoccur the most in the dataset (mean horizontal support)?

KLW Search baseline}

Who is more likely to have an increased appetite, men or women?

PanTeraV Tabular

What are the types of events that occur the most ACROSS patients (vertical support of 100)?

PanTeraV Graphical

Who is more likely to be happier, men or women?

Backward Exploration Questions - Diabetes Dataset

KLW Tabular baseline

Researchers are looking for events that precede an increase in glucose level. They've identified that albumin often increases before glucose increases. Is there another type of event that precedes glucose increase, which is more frequent than the increase in albumin?

Researchers are looking for events that precede an increase in HBA1C levels. They've identified that cholesterol often increases before HBA1C increases. Is there another event type that precedes this sequence of increases (increase in cholesterol, before an increase in HBA1C)?

PanTeraV Tabular

Researchers are interested in the event types that precede an increase in Creatinine levels. They've identified that LDL often increases before Creatinine increases. Is there another event that precedes an increase in Creatinine, which is more frequent that LDL increase?

Researchers are looking for events that precede a period of increase in Cholesterol levels. They've identified that LDL often increases before Cholesterol increases. Is there another event type that precedes this sequence of increases (increase in LDL, before an increase in Cholesterol)?

Backward Exploration Questions - Falls Dataset

KLW Tabular baseline

Researchers are looking for events that precede decreased appetite. They've identified that falling often appears before decreased appetite. Is there another event type that precedes this sequence of events (falling, before decreased appetite)?

PanTeraV Tabular

Researchers are looking for events that precede decreased exercising. They've identified that falling often appears before the decrease in exercising. Is there another event type that precedes this sequence of events (falling, before less exercising)?

Forward Exploration Questions - Diabetes Dataset

- KLW Search baseline
  - Is it more common that
    - a patient has an increase in HBA1C before an increase in Cholesterol?
    - a period of increase in HBA1C after a period of increase in Cholesterol?
- PanTeraV Graphical
  - Is it more common that
    - a patient has a period of decrease in LDL before a period of decrease in glucose?
    - a period of decrease in LDL after a period of decrease in glucose?

Forward Exploration Questions - Falls Dataset

KLW Tabular baseline

Physicians want to examine the relationship between falling and attending fewer social events (recorded as 'Entertainment.Low'). In which of the following two patterns, the period of attending fewer social events is longer?

'low entertainment' then falling

falling then 'low entertainment'

KLW Search baseline

Is it more common that

a person falls and then engage less in entertainment

lose interest in entertainment and then fall

PanTeraV Tabular

Physicians want to examine the relationship between falling and decreased appetite (recorded as 'appetite.medium'). In which of the following two patterns, the period of decreased appetite is longer?

Decreased appetite then falling

Falling then decreased appetite

PanTeraV Graphical

Is it more common that

a person has an increased appetite before falling

after falling having an increased appetite
